# Re-assessing the late HIV diagnosis surveillance definition in the era of increased and frequent testing

**DOI:** 10.1111/hiv.13394

**Published:** 2022-09-07

**Authors:** Peter D Kirwan, Sara Croxford, Adamma Aghaizu, Gary Murphy, Jennifer Tosswill, Alison E Brown, Valerie C Delpech

**Affiliations:** 1 Medical Research Council Biostatistics Unit, University of Cambridge, Cambridge; 2 United Kingdom Health Security Agency, London

**Keywords:** HIV, late diagnosis, late presentation, HIV testing, seroconversion

## Abstract

**Objectives:**

Late HIV diagnosis (CD4<350 cells/mm^3^) is a key public health metric. In an era of more frequent testing there is greater likelihood of HIV diagnosis occurring during seroconversion, when CD4 counts may dip below 350. We apply a correction, considering markers of recent infection and reassess one-year mortality following late diagnosis.

**Methods:**

We used national epidemiological and laboratory surveillance data on all persons diagnosed with HIV in England, Wales, and Northern Ireland (EW&NI). Persons with a baseline CD4<350 were reclassified as “not-late” if they had evidence of recent infection (recency test and/or negative test within 24 months). A correction factor (CF) was the number reclassified divided by the number with CD4<350.

**Results:**

Of the 32,227 people diagnosed with HIV in EW&NI between 2011-2019 with a baseline CD4 (81% of total), 46% had a CD4<350 (uncorrected late diagnosis rate): 34% of gay and bisexual men (GBM), 65% of heterosexual men, and 56% of heterosexual women.

Accounting for recency test and/or prior negative tests gave a “corrected” late diagnosis rate of 39% and corresponding CF of 14%. The CF increased from 10% to 18% during 2011-2015, then plateaued, and was larger among GBM (25%) than heterosexual men and women (6% and 7%, respectively). One-year mortality among persons diagnosed late was 329 per 10,000 after reclassification (an increase from 288/10,000).

**Conclusions:**

The case-surveillance definition of late diagnosis increasingly over-estimates late presentation, the extent of which differs by key populations. Adjustment of late diagnosis is recommended, particularly for frequent testers such as GBM.

## Introduction

CD4 cell count is a key clinical measurement used in assessing a person’s immunological status at the time of HIV diagnosis. CD4 counts among adults without HIV or other immunodeficiency typically range between 500-1500 cells/mm^3^ [1]. Following the initial seroconversion phase, in the absence of treatment, CD4 counts decline to zero following an approximately quadratic trend [2]. This decline has been characterised in several population studies which estimate an HIV diagnosed adult with a CD4 count of 350 cells/mm^3^ to have been living with HIV for 3-5 years, although estimates vary considerably by age, ethnicity, and exposure category [3–7]. Defining thresholds for early and late presentation of HIV according to CD4 count is useful for public health monitoring of testing programmes. The European Late Presenter Consensus working group definition of “late HIV diagnosis” is a CD4<350 cells/mm^3^, or an AIDS-defining illness at presentation [8]. This definition has been adopted by the World Health Organisation and late diagnosis is tracked as a major public health marker by the European Centre for Disease Prevention and Control [9,10], and is a priority area in the HIV Action Plan for England 2022-to-2025 [11].

Late HIV diagnosis has important consequences at both individual and population level and, crucially, is a potentially preventable harm. For the individual, late diagnosis is the most important predictor of morbidity and premature death [12–15] and often results in higher medical costs to the health service [16]. At a population level, late diagnosis suggests missed opportunities to reduce onwards transmission of HIV, by reducing infectivity through anti-retroviral treatment (ART) initiation and/or reducing high-risk behaviour [17,18]. The proportion of people diagnosed late is used as an important public health tool to evaluate the success of HIV testing strategies [19], since a decrease in the number and proportion of people diagnosed late alongside sustained or increased testing volumes indicates a trend towards earlier diagnosis. In the United Kingdom (UK), rates of late HIV diagnosis fell substantially from around 60% in the early 2000s to 42% in 2014, despite increased testing [20]. While rates have since plateaued, the number diagnosed late continues to decline, linked to improvements in testing [10,21].

In the UK, implementation of strategies including test and treat, community point of care testing, self-testing and home-testing, partner notification, as well as campaigns to normalise regular HIV testing, such as European Testing week [22], have led to large increases in regular and repeat testing particularly among gay, bisexual and other men who have sex with men (GBM) who attend sexual health services (SHS) [21,23–26]. Furthermore, 26,000 participants (mostly GBM) registered with the pre-exposure prophylaxis (PrEP) Impact Trial in England accessed 3-monthly testing during 2017-2020 [27]. The success of these combination prevention initiatives over the last decade has increased the likelihood of HIV diagnosis occurring during seroconversion [21]. Of concern, as a result, is that the use of CD4 count at diagnosis to measure population-level late HIV presentation may be increasingly subject to error. It is well known that during the roughly 3-week seroconversion stage CD4 counts are prone to dip; with studies suggesting up to a third of people experience a CD4 count below the 350 cells/mm^3^ threshold during this time [28,29]. An HIV diagnosis occurring during seroconversion could therefore be misclassified as a late presentation. This challenge in correctly determining late diagnosis was highlighted in the 2020 HIV Commission Report [30] and Sasse et al. have previously emphasised the problem of low CD4 counts during seroconversion upon population-level late diagnosis rates [28].

We aimed to quantify to what extent an HIV diagnosis during seroconversion may be misclassified as a late diagnosis among people diagnosed in England, Wales, and Northern Ireland (EW&NI). We present an algorithm which incorporates routine tests for recent infection and evidence of a recent negative HIV test into measurement of population-level late diagnosis rates and define a correction factor which may be applied to populations with similar testing patterns. Finally, we recalculate one-year mortality after reclassification.

## Methods

### Data sources

HIV surveillance data for EW&NI are collected, stored and analysed by the UK Health Security Agency (UKHSA) using the HIV & AIDS Reporting System (HARS), as previously described [31]. Briefly, since 1982, confidential, pseudonymised case-reports of new HIV diagnoses in EW&NI have been collected from a range of settings (including inpatient and outpatient services, general practitioners, community services, and laboratories), in addition to follow-up reports of ongoing HIV care at specialist outpatient clinics. Variables collected include date of UK HIV diagnosis, date and result of baseline CD4 test, date of ART initiation, HIV testing history (i.e. year of last negative test), and other clinical markers.

CD4 data are supplemented by reports received directly from laboratories. HIV testing history is supplemented with information from the GUMCAD sexually transmitted infection (STI) surveillance system, which received reports of all HIV tests at SHS in England since 2009. Laboratory testing of serological samples for recent infection by UKHSA was introduced nationally in 2009, and since 2011 samples have been received from half of all clinics in EW&NI and tested using a biomarker assay (see Appendix for details).

### Record linkage

We linked the HARS, CD4 laboratory surveillance, GUMCAD and serological recency test datasets using deterministic matching algorithms, based on clinic and clinic number, or the combination of Soundex (a four-character coding of the surname [32]), date of birth, gender and other demographic information, using a methodology derived from [33]. The linked data was then deduplicated, retaining the first diagnosis record for each individual. We applied a Recent Infection Testing Algorithm (RITA) to the linked dataset, combining serological recency test results with HIV diagnosis and treatment data (see Appendix and [34]) to obtain classifications of ‘recent’ or ‘not recent’.

### Study population

The study population comprised all adults (aged 15+) first diagnosed in EW&NI between 2011 and 2019 with a baseline CD4 count taken within -14 to 91 days of diagnosis (negative range accounts for diagnosis occurring after sample collection). People reported as first diagnosed with HIV abroad (n=6,745) were excluded from the study as ART history outside the UK, which would alter CD4 progression, was unknown.

### Reclassification methodology

A ‘late’ diagnosis was a baseline CD4 count <350 cells/mm^3^, this definition included most people presenting with an AIDS-defining illness (94%; 1,981/2,098). Adults initially assigned as ‘late’ with evidence of being diagnosed during seroconversion (either a negative HIV test within 24 months of HIV diagnosis, or a ‘recent’ RITA result, or both) were reclassified to ‘not late’ (Box 1).

The correction factor (CF) was the percentage change required for correction of the population-level late diagnosis rate, calculated as: number of diagnoses reclassified as ‘not late’ divided by number of diagnoses within the population of interest. One-year mortality was the number of people who died (all-cause deaths) within 365 days of HIV diagnosis, divided by the total number diagnosed in the same calendar year.

### Statistical methodology

Logistic regression with 95% confidence threshold was used to compare risk factors for reclassification, adjusted and unadjusted odds ratios are provided. Four sensitivity analyses were used to assess the impact of modifying the reclassification algorithm: 1) reducing the previous negative test threshold to 12 months, 2) using only previous negative test information, 3) using only RITA information, 4) restricting the population to the subset with a RITA result.

Data linkage and analysis was performed at UKHSA using Stata 15.1 (StataCorp, College Station, Texas, USA) and R 4.1.0 (R Foundation for Statistical Computing, Vienna, Austria).

## Results

A total of 39,909 adults were first diagnosed with HIV in EW&NI between 2011 and 2019, of whom 81% (32,227) had a baseline CD4 count available. Completeness of baseline CD4 count declined over time, from 88% (4,769/5,408) in 2011 to 73% (2,139/2,943) in 2019. No significant difference by age or country of birth was observed between those with and without CD4 count reported.

Those whose probable route of HIV exposure was injecting drug use, other (vertical transmission or blood contact) or undetermined were less likely to have a CD4 count reported compared to sexual acquisition (average of 74%, 74% and 33%, respectively, vs. 87%) (Supplementary Table 1).

Characteristics of the 32,227 individuals with a baseline CD4 count (the study population) are shown in Table 1. The majority were men (76%) and aged 25-49 years at diagnosis (71%). Around half (53%) had likely acquired HIV through sex between men, 40% through heterosexual sex, 2% through injecting drug use and 1% through another route. Route of exposure was undetermined for 4%. Around half of the study population (47%) were born in the UK, 14% in Europe, 22% in Africa, 13% elsewhere, and 4% had an unknown country of birth. Almost half (44%) were diagnosed in London, 14-19% in each of the other regions of England, and 4% in Northern Ireland or Wales.

A RITA result was available for 55% (17,822/32,227) of the study population, coverage remained above 50% for all years, although was highest during 2014-2017. RITA results were more likely to be available for GBM (58% vs. 40%-53% other exposure groups); younger individuals (58% for those aged 15-24 vs. 48% for those aged 65+), and persons diagnosed in London, the North of England and Northern Ireland (63%, 63% and 80% respectively vs. 54% for South of England, 51% for Midlands and East of England, and 4% for Wales). Result availability did not differ greatly on other measures.

**Figure 1:**
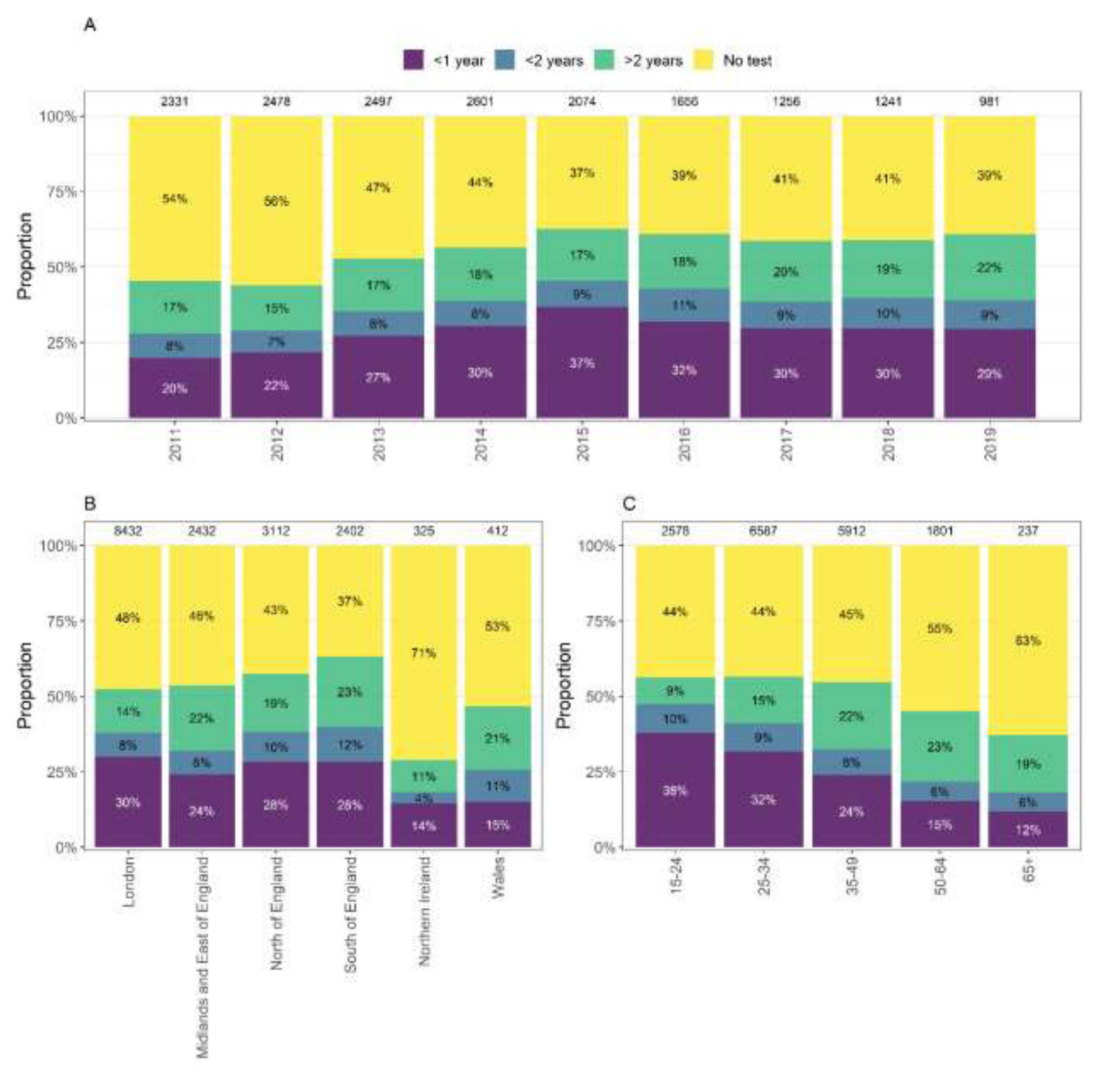
Availability of last HIV negative test information for gay and bisexual men diagnosed with HIV between 2011-2019, by (A) year of diagnosis, (B) region of diagnosis and (C) age at diagnosis Footnotes: 1. Total number of diagnoses by each year and exposure group shown at top of bar.

Evidence for at least one previous negative HIV test was available for 41% (13,180/32,227) of individuals, this increased from 33% (1,566/4,769) in 2011 to 46% (975/2,139) in 2019. Testing history was more commonly available for GBM (54% vs. 11%-34% others) and younger people (48% for those aged 15-24 vs. 20% for those aged 65+). Among GBM, those diagnosed in the South and North of England were most likely to have previous negative tests reported (63% and 57%), and testing history was more commonly reported for younger GBM: 56% among those aged 15-24 or 25-34 at diagnosis vs. 37% for GBM aged 65+ (Figure 1).

Overall, 71% (22,993/32,227) of individuals had either a RITA result or evidence of a previous negative HIV test (Supplementary Table 1).

### Reclassification

The proportion of the study population with a baseline CD4<350 cells/mm^3^ (i.e. late diagnosis rate without correction) was 46% (14,803/32,227). This proportion reduced from 50% in 2011 to 42% in 2015, then increased to 49% in 2019, despite a declining trend in cases over the period. Among those with a CD4<350 cells/mm^3^, 4% (612/14,803) had a RITA result indicating recent HIV acquisition, 12% (1,771/14,803) had a negative test within 24 months, and 14% (2,088/14,803) had at least one of these. The CF was therefore calculated as 14% overall. The CF increased from 10% in 2011, to 18% in 2015 and plateaued around 15% in 2016-2019. After correction, 39% of people (12,715/32,227) were classified as late diagnoses (Table 2).

**Figure 2:**
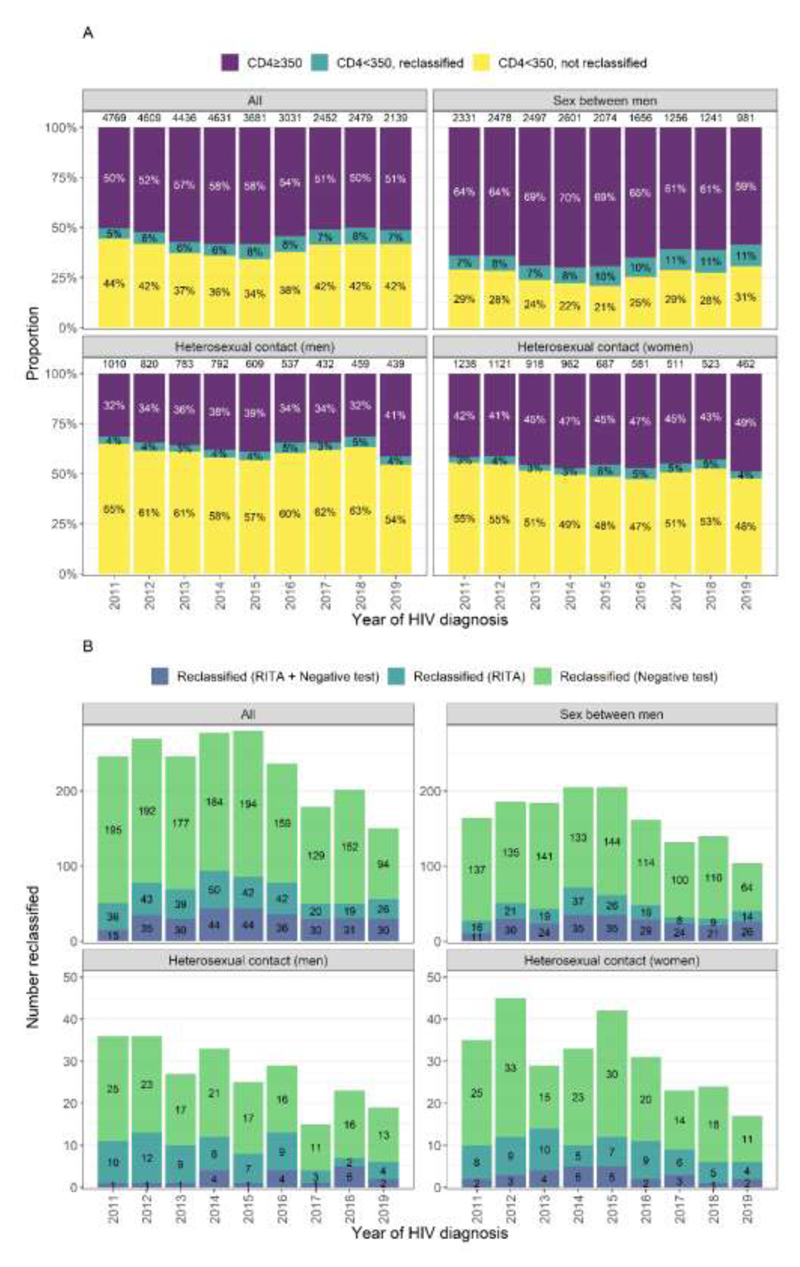
HIV diagnoses by year of HIV diagnosis, route of HIV exposure, CD4 count, re-classification (panel A) and reason for re-classification (panel B) Footnotes: 1. Total number of diagnoses by each year and exposure group shown at top of bar in panel A. 2. Note differing axes in panel B.

Figure 2A shows the proportion of individuals diagnosed with CD4≥350 cells/mm^3^ and CD4<350 cells/mm^3^, by reclassification and probable route of HIV exposure. The evidence used to reclassify are shown in Figure 2B. Among GBM the proportion with a CD4<350 cells/mm^3^ fell from 36% in 2011 to 30% in 2014, rising to 41% in 2019, this compared to a relatively steady reduction among heterosexual men (from 68% in 2011 to 59% in 2019) and women (from 58% in 2011 to 51% in 2019). The number and proportion reclassified was greater among GBM than heterosexual men and women, reflected in an overall CF of 25% among GBM compared to 6% and 7% respectively (Tables 3-5).

Among GBM the CF rose from 20% in 2011 to 32% in 2015, stabilising at around 27% in 2016-2019. The CF was highest among younger GBM (46% for those aged 15-24 compared to 10% for 65+) and elevated for GBM diagnosed in London (30%) as compared to other parts of EW&NI (range 14-25%) (Table 3).

For heterosexual men, the CF grew from 5% in 2011 to 8% in 2016, remaining around 7% in subsequent years. For heterosexual women these figures were 5% in 2011, 11% in 2015 and around 8% in subsequent years (Tables 4-5). The CF was higher among younger heterosexual men and women (18% and 22% respectively for those aged 15-24, compared to 6% and 3% for ages 65+) and marginally higher in London (8%) compared to the rest of EW&NI (2-7%).

**Figure 3:**
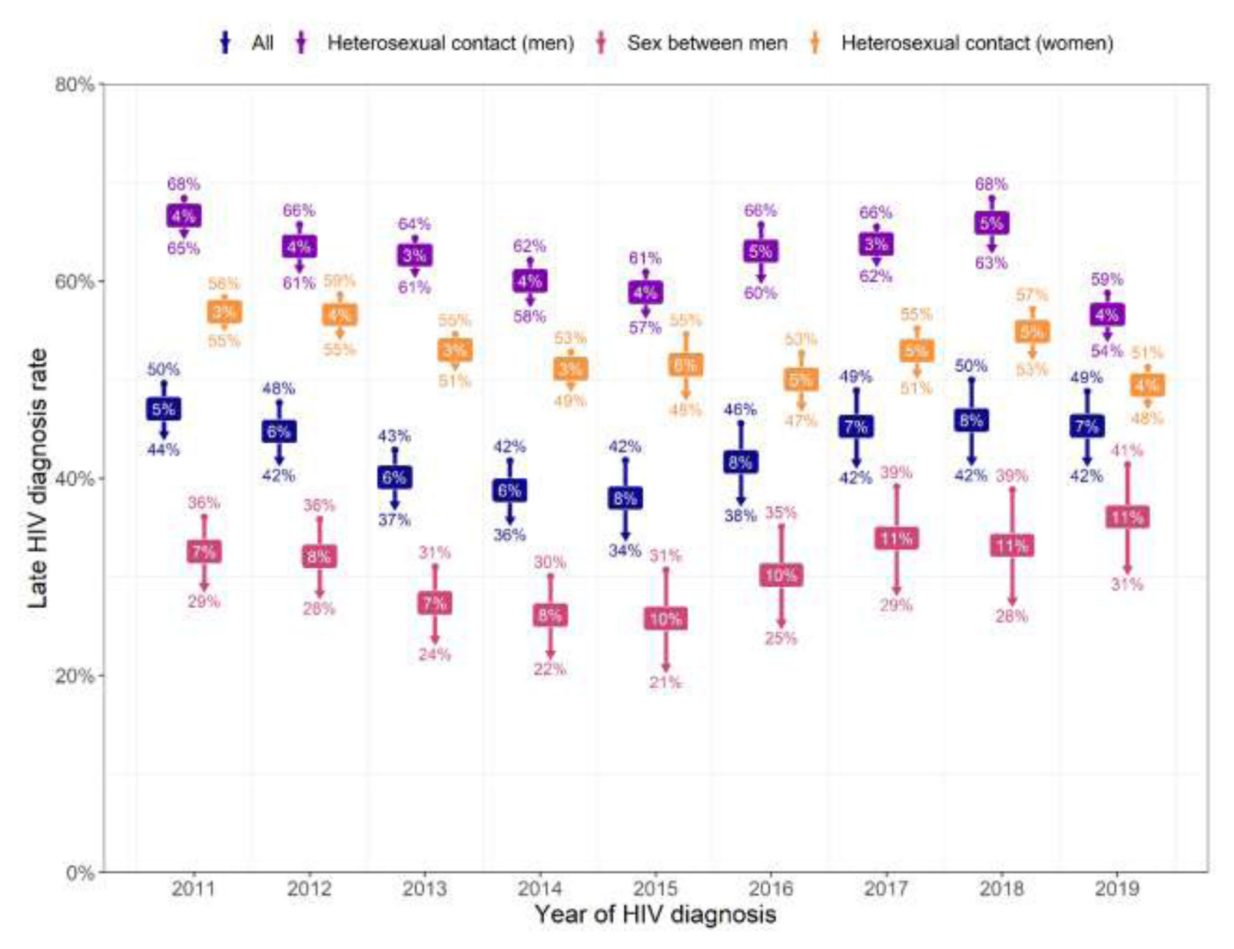
Absolute change^1^ in late diagnosis rate following reclassification, by route of exposure and year of HIV diagnosis, 2011-2019 Footnotes: 1. Upper value is late diagnosis rate before reclassification, lower value is late diagnosis rate after reclassification, labels are absolute change in late diagnosis rate.

Figure 3 depicts the absolute change in late diagnosis rate following reclassification. This increased over time among GBM (from 7% in 2011 to 11% in 2019), remaining relatively stable for heterosexual men and women (between 3-6%).

### Sensitivity analyses

By reducing the threshold for a last negative test to 12 months, 553 fewer diagnoses were reclassified, resulting in a CF of 10% and late diagnosis rate of 41% (vs. CF of 14% and 39% late diagnosed using the 24-month threshold). Among GBM, the shorter threshold resulted in a CF of 19% (vs. 25%), and among heterosexual men and women of 5% and 5% (vs. 6% and 7%) (Supplementary Table 2).

Using solely a last negative test within the past 24 months to reclassify diagnoses resulted in a CF of 12% overall and late diagnosis rate of 40%. The CF was 22% for GBM, 5% for heterosexual men, and 6% for heterosexual women. Conversely, using solely RITA information resulted in CF of 4% overall, 7% for GBM, and 2% for both heterosexual men and women (Supplementary Table 2).

A total of 17,822 people had both a baseline CD4 count and RITA result reported. In this group 46% (8,198) had a CD4<350 cells/mm^3^, of whom 7% (612/8,189) had a RITA result indicating recent acquisition, 13% (1,092/8,189) had a negative test within 24 months, and 17% (1,409/8,189) had at least one of these. After reclassification the late diagnosis rate was 38% overall, with a CF of 17%. The CF was 29% for GBM, 8% for heterosexual men, and 9% for heterosexual women (Supplementary Table 2).

### Risk factors for reclassification

**Figure 4:**
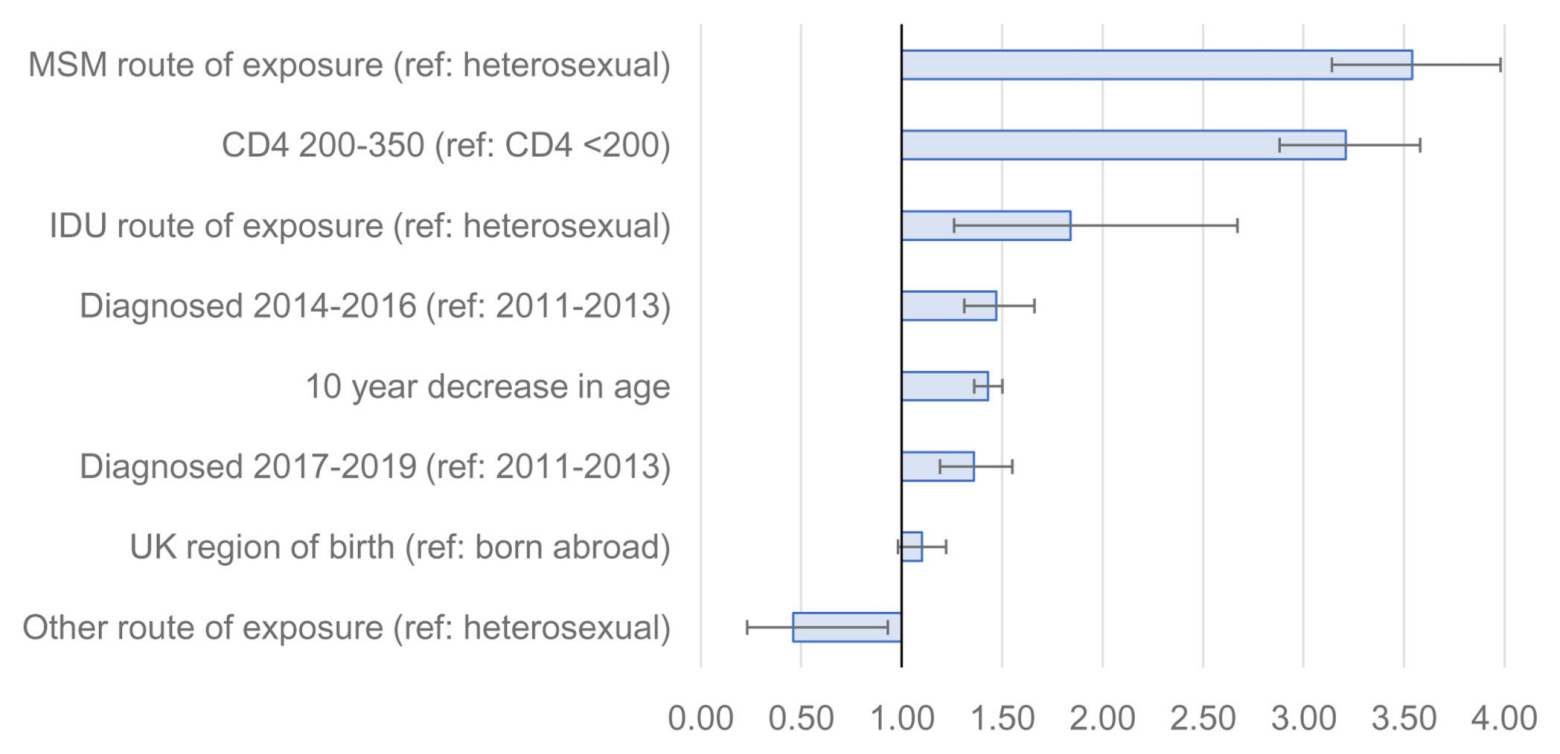
Adjusted odds ratios and 95% confidence intervals for reclassification of late diagnosis among people diagnosed with a CD4 count <350 cells/mm^3^

In multivariable analysis adjusted for other factors, the odds of being reclassified for GBM were three times those of heterosexual men and women (aOR 3.54, 95% CI 3.14-3.98, P<0.001) and the odds for those with a baseline CD4 between 200-350 cells/mm^3^ were three times those with a CD4<200 cells/mm^3^ (aOR 3.21, 95% CI 2.88-3.58, P<0.001). A younger age at diagnosis (aOR 1.43, 95% CI 1.36-1.50, P<0.001 for each 10-year decrease in age) and more recent diagnosis (2014-16 and 2017-19 vs. 2011-13) were also significant factors for reclassification (Table 6/Figure 4). There was an approximately linear relationship between increasing CD4 count and the proportion of diagnoses reclassified (Supplementary Figure 2).

### One-year mortality

Considering individuals with a baseline CD4 count and at least one year of follow-up, 158 per 10,000 population (447/30,088) diagnosed between 2011-2018 died within a year of diagnosis, of whom 89% (n=396) were diagnosed with a CD4<350 cells/mm^3^. The one-year mortality rate prior to correction was 288 per 10,000 for people with a CD4<350 cells/mm^3^, compared to 31.2 per 10,000 with a CD4≥350 cells/mm^3^. Following reclassification, these rates were 329 and 31.8 per 10,000 (36.1 per 10,000 among those reclassified) (Table 7). All 7 reclassified individuals who died had non-HIV related causes of death. Mortality rates did not change substantially over the study period.

## Discussion

The success of combination prevention initiatives including testing campaigns [23], immediate ART initiation [35], and use of PrEP by those most at risk [36] is evident in estimated declines in incident cases and estimated reductions in undiagnosed HIV prevalence in England since 2013 [37,38]. The proportion of people diagnosed late each year has been an important surveillance output used to direct and target these HIV prevention campaigns and late diagnosis is a key indicator of the English Public Health Outcome Framework; presented by key exposure group and geography on the fingertips mapping tool [39].

In this study, we reassessed the surveillance definition of late diagnosis in the context of people testing more frequently for HIV, leading to higher likelihood of being diagnosed during seroconversion. Our analyses indicate that an increasing proportion of people diagnosed with HIV have evidence of a recent negative HIV test. When testing history is combined with knowledge of recent infection using RITA we show that the current public health definition of late diagnosis (CD4<350 cells/mm^3^) increasingly over-estimates rates of late presentation. Reclassification for seroconversion resulted in a downwards adjustment of the late diagnosis rate by 14% in 2019. This was highest among GBM (26% in 2019), particularly younger GBM (CF of 46% for those aged 15-24) and those living in London (CF of 30%), which may reflect the concentration of HIV prevention campaigns and high testing volumes at SHS (and therefore higher likelihood of being diagnosed during seroconversion) in this age group and region compared to other areas of the country [40–42]. Meanwhile, groups for whom reclassification was less common (e.g. older people and those who likely acquired HIV through heterosexual sex) are known to have lower testing rates [21].

For GBM, the CF increased between 2011 and 2015 (from 20% to 32%), declining to around 27% in more recent years despite high testing rates being maintained. The recent decline may reflect the reduction in HIV incidence among GBM estimated in recent years [37]. The knock-on effect is an upward trend in late diagnosis rates (from 21% in 2015 to 31% in 2019, after accounting for seroconversion). We surmise that fewer HIV infections acquired (e.g. due to PrEP) led to a smaller population able to be diagnosed at an early stage, thereby shrinking the denominator for the late diagnosis indicator more rapidly than the numerator. More modest declines in late diagnosis rates were observed for heterosexuals. In contrast to GBM, frequent testing and uptake of PrEP among higher risk heterosexuals has not been observed [21,43], hence fewer heterosexuals were diagnosed during seroconversion resulting in a comparatively low CF. Trends in late diagnosis rates must be interpreted alongside the absolute number of diagnoses, as well as estimated incidence, testing, and PrEP use.

In an era of effective treatments, late presentation of HIV is the strongest predictor of premature death [14, 15]. In our study, people diagnosed with a CD4<350 cells/mm^3^ had a nine-fold increased risk of death within a year of diagnosis compared to those with a CD4≥350 cells/mm^3^, which increased to 10-fold after reclassification.

Sasse et al. previously used evidence of recent acquisition within 6 months, clinical presentation with acute infection, and/or recent risk behaviour with a HIV-positive partner to reclassify late diagnoses [28]. While less stringent, we consider our definition more appropriate for EW&NI as it includes less frequent testers and those without testing history. For people with both a recent RITA and testing history, the median time from previous negative test to diagnosis was 6 months, with 86% of previous negative tests within 24 months. Use of testing history alone could be a pragmatic approach to correct for seroconversion in countries lacking detailed clinical or serological information. Where collected, information from diagnostic tests could also provide a useful proxy for recency, recent studies have shown correlation between a ‘recent’ LAg result and a signal-to-cutoff ratio <250 in the widely-used Abbott Architect Ag/Ab assay [44,45].

The strengths of this study are the very high coverage of HIV diagnoses, high completeness of key variables, and long period over which data was able to be linked. A limitation was differential geographical coverage of RITA results, which may have biased reclassification towards certain regions. Additionally, HIV testing history outside SHS was poorly captured; whereas GBM are more likely to attend SHS, heterosexual men and women may access testing in a wider variety of settings [21].

In this study we focus on those with a first diagnosis in EW&NI as we could not capture ART initiation or engagement with care prior to arrival. In 2019, 72% of those previously diagnosed abroad were virally suppressed, with only 22% having a CD4<350 cells/mm^3^ at first presentation in EW&NI (compared to 49% for the study population). Whilst this group was not included in our analysis, the health needs of migrants remain a public health priority, especially the potential for disengagement in care following re-location.

## Conclusions

As highlighted in the recent English HIV Action Plan [11], late diagnosis remains a priority area in HIV case-reporting surveillance. It provides an indicator of a ‘system failure’ to promptly diagnose, provide benefit through ART, and reduce onward transmission, and has the advantage of being relatively easy to measure. Tackling barriers which may lead to late presentation: testing access, lack of risk awareness, and HIV-related stigma is vital to reduce the number of late diagnoses [46,47].

Our refined definition of late HIV diagnosis focuses this measure on those at greatest risk of mortality. This is ever-more important as the overall number of diagnoses reduces, since unadjusted rates may present a misleading picture of where late diagnoses are occurring, undermining targeted interventions tailored towards late diagnosis. This refinement is also key for detailed incident follow-up of individual cases and may contribute to the development of statistical models which use CD4 count information to estimate time since infection [7,37,48]. For example, inclusion of RITA and/or last negative test information as biomarkers in these models may aid model performance and help to address misclassification of long-standing infection.

## Supplementary Material

Supplementary File

## Data Availability

The data used in this study are protected data. These data are not publicly available because the information is personal or special category personal data, and there is risk of ‘re-identification’ of data that has been anonymised by data matching, inference or deductive disclosure. Access to protected data is subject to robust governance protocols, where it is lawful, ethical and safe to do so. Individuals and organisations wishing to request access to data used in this study, can make a request directly to UKHSA (https://www.gov.uk/government/publications/accessing-ukhsa-protected-data). Access to protected data is always strictly controlled using legally binding data sharing contracts.

## Box 1 Revised definition of late HIV diagnosis

Among adults (aged 15+) with a baseline CD4 count (within -14 to 91 days), those with a CD4 count <350 cells/mm^3^ and neither:

(a) a RITA result indicating recent HIV acquisition;
(b) a negative HIV test result within the preceding 24 months.
