# Re‐assessing the late HIV diagnosis surveillance definition in the era of increased and frequent testing

**DOI:** 10.1111/hiv.13394

**Published:** 2022-09-07

**Authors:** Peter D. Kirwan, Sara Croxford, Adamma Aghaizu, Gary Murphy, Jennifer Tosswill, Alison E. Brown, Valerie C. Delpech

**Affiliations:** ^1^ United Kingdom Health Security Agency London UK; ^2^ Medical Research Council Biostatistics Unit University of Cambridge Cambridge UK

**Keywords:** HIV, HIV testing, late diagnosis, late presentation, seroconversion

## Abstract

**Objectives:**

Late HIV diagnosis (CD4 <350 cells/mm^3^) is a key public health metric. In an era of more frequent testing, the likelihood of HIV diagnosis occurring during seroconversion, when CD4 counts may dip below 350, is greater. We applied a correction, considering markers of recent infection, and re‐assessed 1‐year mortality following late diagnosis.

**Methods:**

We used national epidemiological and laboratory surveillance data from all people diagnosed with HIV in England, Wales, and Northern Ireland (EW&NI). Those with a baseline CD4 <350 were reclassified as ‘not late’ if they had evidence of recent infection (recency test and/or negative test within 24 months). A correction factor (CF) was the number reclassified divided by the number with a CD4 <350.

**Results:**

Of the 32 227 people diagnosed with HIV in EW&NI between 2011 and 2019 with a baseline CD4 (81% of total), 46% had a CD4 <350 (uncorrected late diagnosis rate): 34% of gay and bisexual men (GBM), 65% of heterosexual men, and 56% of heterosexual women.

Accounting for recency test and/or prior negative tests gave a ‘corrected’ late diagnosis rate of 39% and corresponding CF of 14%. The CF increased from 10% to 18% during 2011–2015, then plateaued, and was larger among GBM (25%) than heterosexual men and women (6% and 7%, respectively). One‐year mortality among people diagnosed late was 329 per 10 000 after reclassification (an increase from 288/10 000).

**Conclusions:**

The case‐surveillance definition of late diagnosis increasingly overestimates late presentation, the extent of which differs by key populations. Adjustment of late diagnosis is recommended, particularly for frequent testers such as GBM.

## INTRODUCTION

CD4 cell count is a key clinical measurement used in assessing a person's immunological status at the time of HIV diagnosis. CD4 counts among adults without HIV or other immunodeficiency typically range between 500 and 1500 cells/mm^3^ [1]. Following the initial seroconversion phase, in the absence of treatment, CD4 counts decline to zero following an approximately quadratic trend [2]. This decline has been characterized in several population studies, which estimate an HIV diagnosed adult with a CD4 count of 350 cells/mm^3^ to have been living with HIV for 3–5 years, although estimates vary considerably by age, ethnicity, and exposure category [3, 4, 5, 6, 7]. Defining thresholds for early and late presentation of HIV according to CD4 count is useful for public health monitoring of testing programmes. The European Late Presenter Consensus working group definition of ‘late HIV diagnosis’ is a CD4 <350 cells/mm^3^ or an AIDS‐defining illness at presentation [8]. This definition has been adopted by the World Health Organization, and late diagnosis is tracked as a major public health marker by the European Centre for Disease Prevention and Control [9, 10] and is a priority area in the HIV Action Plan for England 2022 to 2025 [11].

Late HIV diagnosis has important consequences for individuals and at the population level and, crucially, is a potentially preventable harm. For the individual, late diagnosis is the most important predictor of morbidity and premature death [12, 13, 14, 15] and often results in higher medical costs to the health service [16]. At a population level, late diagnosis suggests missed opportunities to reduce onwards transmission of HIV by reducing infectivity through antiretroviral treatment (ART) initiation and/or reducing high‐risk behaviour [17, 18]. The proportion of people diagnosed late is used as an important public health tool to evaluate the success of HIV testing strategies [19], since a decrease in the number and proportion of people diagnosed late alongside sustained or increased testing volumes indicates a trend towards earlier diagnosis. In the UK, rates of late HIV diagnosis fell substantially from around 60% in the early 2000s to 42% in 2014, despite increased testing [20]. Rates have since plateaued, but the number diagnosed late continues to decline, which is linked to improvements in testing [10, 21].

In the UK, implementation of strategies including test and treat, community point‐of‐care testing, self‐testing and home testing, partner notification, and campaigns to normalize regular HIV testing, such as European Testing week [22], have led to large increases in regular and repeat testing, particularly among gay, bisexual, and other men who have sex with men (GBM) who attend sexual health services (SHS) [21, 23, 24, 25, 26]. Furthermore, 26 000 participants (mostly GBM) who were registered with the pre‐exposure prophylaxis (PrEP) Impact Trial in England accessed 3‐monthly testing during 2017–2020 [27]. The success of these combination prevention initiatives over the last decade has increased the likelihood of HIV diagnosis occurring during seroconversion [21]. Of concern, as a result, the use of CD4 count at diagnosis to measure population‐level late HIV presentation may be increasingly subject to error. It is well known that, during the roughly 3‐week seroconversion stage, CD4 counts are prone to dip; studies suggest that up to one‐third of people experience a CD4 count below the 350 cells/mm^3^ threshold during this time [28, 29]. An HIV diagnosis occurring during seroconversion could therefore be misclassified as a late presentation. This challenge in correctly determining late diagnosis was highlighted in the 2020 HIV Commission Report [30], and Sasse et al. previously emphasized the problem of low CD4 counts during seroconversion upon population‐level late diagnosis rates [28].

We aimed to quantify the extent to which an HIV diagnosis during seroconversion may be misclassified as a late diagnosis among people diagnosed in England, Wales, and Northern Ireland (EW&NI). We present an algorithm that incorporates routine tests for recent infection and evidence of a recent negative HIV test into measurement of population‐level late diagnosis rates and define a correction factor that may be applied to populations with similar testing patterns. Finally, we recalculate 1‐year mortality after reclassification.

## METHODS

### Data sources

HIV surveillance data for EW&NI are collected, stored, and analysed by the UK Health Security Agency (UKHSA) using the HIV & AIDS Reporting System (HARS), as previously described [31]. Briefly, since 1982, confidential, pseudonymised case reports of new HIV diagnoses in EW&NI have been collected from a range of settings (including inpatient and outpatient services, general practitioners, community services, and laboratories) in addition to follow‐up reports of ongoing HIV care at specialist outpatient clinics. Variables collected include date of UK HIV diagnosis, date and result of baseline CD4 test, date of ART initiation, HIV testing history (i.e. year of last negative test), and other clinical markers.

CD4 data are supplemented by reports received directly from laboratories. HIV testing history is supplemented with information from the Genitourinary Medicine Clinic Activity Dataset (GUMCAD) sexually transmitted infection surveillance system, which received reports of all HIV tests at SHS in England since 2009. Laboratory testing of serological samples for recent infection by UKHSA was introduced nationally in 2009; since 2011, samples have been received from half of all clinics in EW&NI and tested using a biomarker assay (see Appendix S1 for details).

### Record linkage

We linked the HARS, CD4 laboratory surveillance, GUMCAD, and serological recency test datasets using deterministic matching algorithms based on clinic and clinic number or the combination of Soundex (a four‐character coding of the surname [32]), date of birth, sex, and other demographic information, using a methodology derived from Winter et al. [33]. The linked data were then deduplicated, retaining the first diagnosis record for each individual. We applied a recent infection testing algorithm (RITA) to the linked dataset, combining serological recency test results with HIV diagnosis and treatment data (see Appendix S1 and [34]) to obtain classifications of ‘recent’ or ‘not recent’.

### Study population

The study population comprised all adults (aged ≥15 years) first diagnosed in EW&NI between 2011 and 2019 with a baseline CD4 count taken within −14 to 91 days of diagnosis (negative range accounts for diagnosis occurring after sample collection). People reported as first diagnosed with HIV abroad (*n* = 6745) were excluded from the study because their ART history outside the UK, which would alter CD4 progression, was unknown.

### Reclassification methodology

A ‘late’ diagnosis was a baseline CD4 count <350 cells/mm^3^; this definition included most people presenting with an AIDS‐defining illness (94% [1981/2098]). Adults initially assigned as ‘late’ with evidence of being diagnosed during seroconversion (either a negative HIV test within 24 months of HIV diagnosis or a ‘recent’ RITA result or both) were reclassified to ‘not late’ (Box 1).

BOX 1 **Revised definition of late HIV diagnosis**

Among adults (aged ≥15 years) with a baseline CD4 count (within −14 to 91 days), those with a CD4 count <350 cells/mm^3^ and neither:

(a) a recent infection testing algorithm (RITA) result indicating recent HIV acquisition nor

(b) a negative HIV test result within the preceding 24 months.

The correction factor (CF) was the percentage change required for correction of the population‐level late diagnosis rate, calculated as the number of diagnoses reclassified as ‘not late’ divided by number of diagnoses within the population of interest. One‐year mortality was the number of people who died (all‐cause deaths) within 365 days of HIV diagnosis, divided by the total number diagnosed in the same calendar year.

### Statistical methodology

We used logistic regression with 95% confidence threshold to compare risk factors for reclassification; adjusted and unadjusted odds ratios are provided. We used four sensitivity analyses to assess the impact of modifying the reclassification algorithm: (1) reducing the previous negative test threshold to 12 months, (2) using only previous negative test information, (3) using only RITA information, and (4) restricting the population to the subset with a RITA result.

Data linkage and analysis was performed at UKHSA using Stata 15.1 (StataCorp, College Station, Texas, USA) and R 4.1.0 (R Foundation for Statistical Computing, Vienna, Austria). Figures were generated using the open‐source R package ggplot2 [35].

## RESULTS

A total of 39 909 adults were first diagnosed with HIV in EW&NI between 2011 and 2019, of whom 81% (32 227) had a baseline CD4 count available. Completeness of baseline CD4 count declined over time, from 88% (4769/5408) in 2011 to 73% (2139/2943) in 2019. No significant difference by age or country of birth was observed between those with and without CD4 count reported. Those whose probable route of HIV exposure was injecting drug use, other (vertical transmission or blood contact), or undetermined were less likely to have a CD4 count reported than were those with sexual acquisition (average of 74%, 74%, and 33% vs. 87%, respectively) (Table S1).

Characteristics of the 32 227 individuals with a baseline CD4 count (the study population) are shown in Table 1. The majority were men (76%) and aged 25–49 years at diagnosis (71%). Around half (53%) had likely acquired HIV through sex between men, 40% through heterosexual sex, 2% through injecting drug use, and 1% through another route (Figure S3). Route of exposure was undetermined for 4%. Around half of the study population (47%) were born in the UK, 14% in Europe, 22% in Africa, and 13% elsewhere; 4% had an unknown country of birth. Almost half (44%) were diagnosed in London, 14%–19% in each of the other regions of England, and 4% in Northern Ireland or Wales.

**TABLE 1:** Study population stratified by availability and result of RITA test and availability and time since last negative HIV test, by year of HIV diagnosis, sex, probable route of HIV exposure, country of birth, age at HIV diagnosis, and region of diagnosis

	Study population^a^	RITA result available		RITA indicates recent infection		Negative HIV test available		Negative HIV test within 12 months		Negative HIV test within 24 months	
	*n*	*n*	%	*n*	%	*n*	%	*n*	%	*n*	%
All years	32 227	17 822	55	3734	12	13 180	41	5671	18	7681	24
Year of HIV diagnosis											
2011	4769	2606	55	397	8	1566	33	563	12	815	17
2012	4609	2347	51	418	9	1543	33	636	14	878	19
2013	4436	2353	53	528	12	1754	40	757	17	1028	23
2014	4631	2688	58	683	15	1986	43	918	20	1197	26
2015	3681	2224	60	511	14	1747	47	878	24	1134	31
2016	3031	1799	59	410	14	1419	47	636	21	868	29
2017	2452	1372	56	296	12	1099	45	464	19	617	25
2018	2479	1308	53	246	10	1091	44	444	18	623	25
2019	2139	1125	53	245	11	975	46	375	18	521	24
Sex											
Male	24 546	13 748	56	3324	14	10 971	45	5195	21	6912	28
Female	7676	4073	53	410	5	2206	29	476	6	767	10
Probable route of HIV exposure											
Sex between men	17 115	9939	58	2890	17	9305	54	4787	28	6269	37
Heterosexual men	5881	3141	53	341	6	1398	24	305	5	504	9
Heterosexual women	7003	3781	54	385	5	2088	30	448	6	710	10
Injecting drug use	554	259	47	35	6	186	34	76	14	102	18
Other	323	129	40	13	4	37	11	5	2	13	4
Undetermined	1351	573	42	70	5	166	12	50	4	83	6
Country of birth											
UK born	15 056	8584	57	2155	14	7307	49	3312	22	4481	30
Rest of Europe	4539	2547	56	670	15	1974	43	1021	22	1339	29
Africa	7140	3829	54	286	4	1941	27	439	6	675	9
Rest of World	4196	2349	56	522	12	1723	41	797	19	1050	25
Unknown	1296	513	40	101	8	235	18	102	8	136	10
Age at HIV diagnosis											
15–24	3816	2211	58	699	18	1814	48	1131	30	1473	39
25–34	10 396	5907	57	1541	15	4958	48	2424	23	3217	31
35–49	12 474	6763	54	1120	9	4896	39	1716	14	2385	19
50–64	4773	2572	54	339	7	1356	28	363	8	544	11
65+	768	369	48	35	5	156	20	37	5	62	8
Region of diagnosis											
London	14 215	8470	60	1966	14	5958	42	2884	20	3764	26
Midlands and East of England	6200	3002	48	528	9	2221	36	803	13	1125	18
North of England	5826	3503	60	649	11	2423	42	1029	18	1413	24
South of England	4622	2391	52	511	11	2211	48	829	18	1185	26
Northern Ireland	580	433	75	72	12	112	19	49	8	63	11
Wales	784	23	3	8	1	255	33	77	10	131	17

Abbreviations: RITA, recent infection testing algorithm.

^a^ Study population are individuals with a baseline CD4 count available.

A RITA result was available for 55% (17 822/32 227) of the study population; coverage remained above 50% for all years but was highest during 2014–2017. RITA results were more likely to be available for GBM (58% vs. 40%–53% other exposure groups); younger individuals (58% for those aged 15–24 vs. 48% for those aged ≥65 years), and people diagnosed in London, the North of England, and Northern Ireland (63%, 63%, and 80% respectively vs. 54% for South of England, 51% for Midlands and East of England, and 4% for Wales). Result availability did not differ greatly on other measures.

**FIGURE 1:**
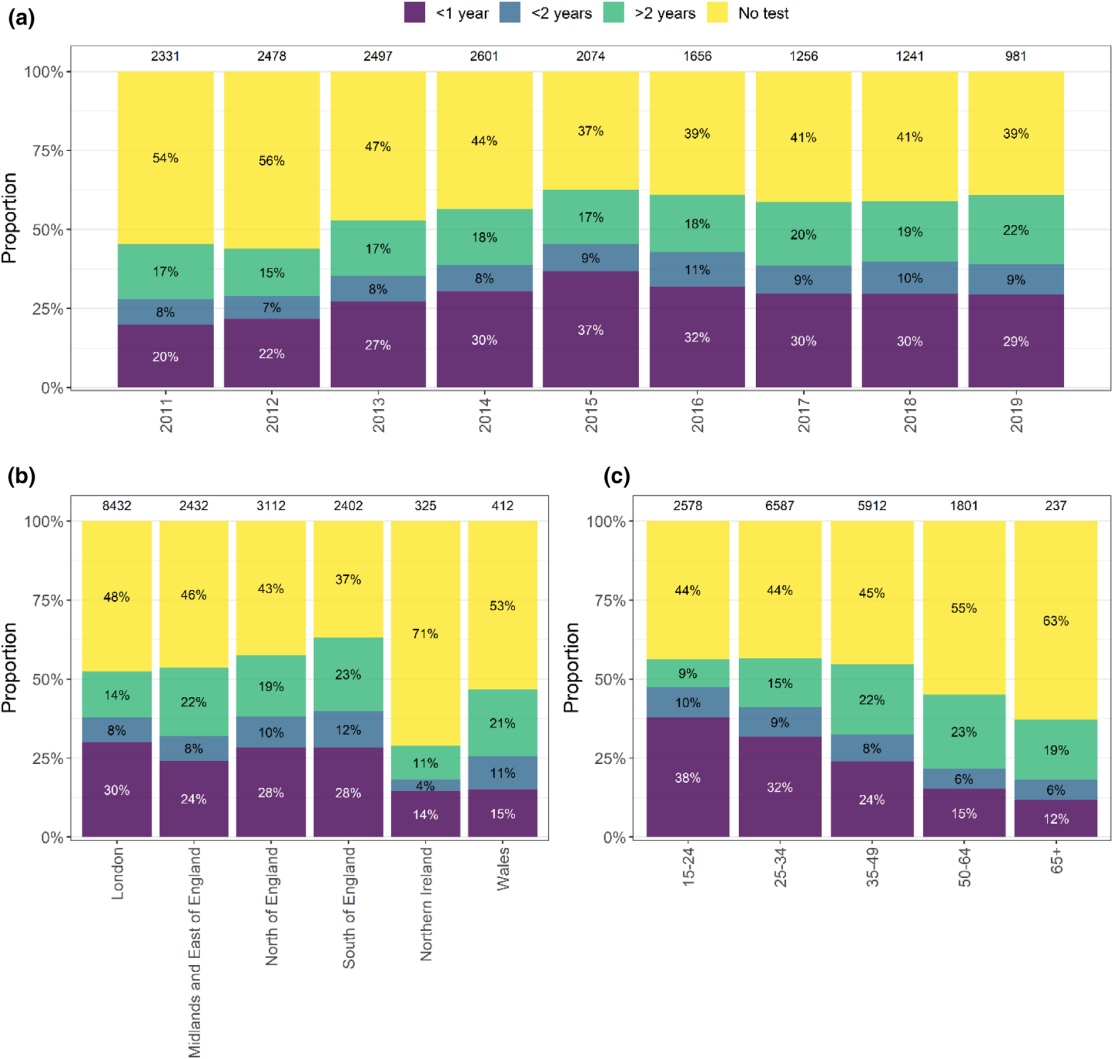
Availability of last HIV‐negative test information for gay and bisexual men diagnosed with HIV between 2011 and 2019, by (a) year of diagnosis, (b) region of diagnosis and (c) age at diagnosis. Total number of diagnoses by each year and exposure group shown at top of bar

Evidence for at least one previous negative HIV test was available for 41% (13 180/32 227) of individuals, which increased from 33% (1566/4769) in 2011 to 46% (975/2139) in 2019. Testing history was more commonly available for GBM (54% vs. 11%–34% others) and younger people (48% for those aged 15–24 vs. 20% for those aged ≥65 years). Among GBM, those diagnosed in the South and North of England were most likely to have previous negative tests reported (63% and 57%), and testing history was more commonly reported for younger GBM: 56% among those aged 15–24 or 25–34 years at diagnosis vs. 37% for GBM aged ≥65 years (Figure 1).

Overall, 71% (22 993/32 227) of individuals had either a RITA result or evidence of a previous negative HIV test (Table S1).

### Reclassification

The proportion of the study population with a baseline CD4 <350 cells/mm^3^ (i.e. late diagnosis rate without correction) was 46% (14 803/32 227). This proportion reduced from 50% in 2011 to 42% in 2015, then increased to 49% in 2019, despite a declining trend in cases over the period. Among those with a CD4 <350 cells/mm^3^, 4% (612/14 803) had a RITA result indicating recent HIV acquisition, 12% (1771/14 803) had a negative test within 24 months, and 14% (2088/14 803) had at least one of these. The CF was therefore calculated as 14% overall. The CF increased from 10% in 2011 to 18% in 2015 and plateaued around 15% in 2016–2019. After correction, 39% of people (12 715/32 227) were classified as late diagnoses (Table 2).

**TABLE 2:** Late diagnosis rates before and after correction, and correction factors, for all individuals with baseline CD4 count, by year of diagnosis, age group, and region of diagnosis

	Total	CD4 <350	CD4 <350 and recent RITA	CD4 <350 and negative test within 24 months	Total reclassified^a^	Late diagnosis rate before correction^b^ (%)	Late diagnosis rate after correction^c^ (%)	Correction factor^d^ (%)
All years	32 227	14 803	612	1771	2088	46	39	14
Year of diagnosis								
2011	4769	2366	51	210	246	50	44	10
2012	4609	2198	78	227	270	48	42	12
2013	4436	1902	69	207	246	43	37	13
2014	4631	1935	94	228	278	42	36	14
2015	3681	1539	86	238	280	42	34	18
2016	3031	1381	78	195	237	46	38	17
2017	2452	1199	50	159	179	49	42	15
2018	2479	1239	50	183	202	50	42	16
2019	2139	1044	56	124	150	49	42	14
Age group								
15–24	3816	1135	113	337	385	30	20	34
25–34	10 396	3917	231	694	805	38	30	21
35–49	12 474	6325	176	554	650	51	45	10
50–64	4773	2906	79	166	218	61	56	8
65+	768	520	13	20	30	68	64	6
Region of diagnosis								
London	14 215	5692	299	851	995	40	33	17
Midlands and East of England	6200	3272	94	283	329	53	47	10
North of England	5826	2856	134	333	411	49	42	14
South of England	4622	2287	71	258	296	49	43	13
Wales/Northern Ireland	1364	696	14	46	57	51	47	8

Abbreviation: RITA, recent infection testing algorithm.

^a^ Total reclassified = CD4 <350 cells/mm^3^ and recent RITA or negative test within 24 months.

^b^ Late diagnosed before correction = CD4 <350 cells/mm^3^/total.

^c^ Late diagnosed after correction = (CD4 <350 cells/mm^3^ − Total reclassified)/total.

^d^ Correction factor = total reclassified/CD4 <350 cells/mm^3^.

**FIGURE 2:**
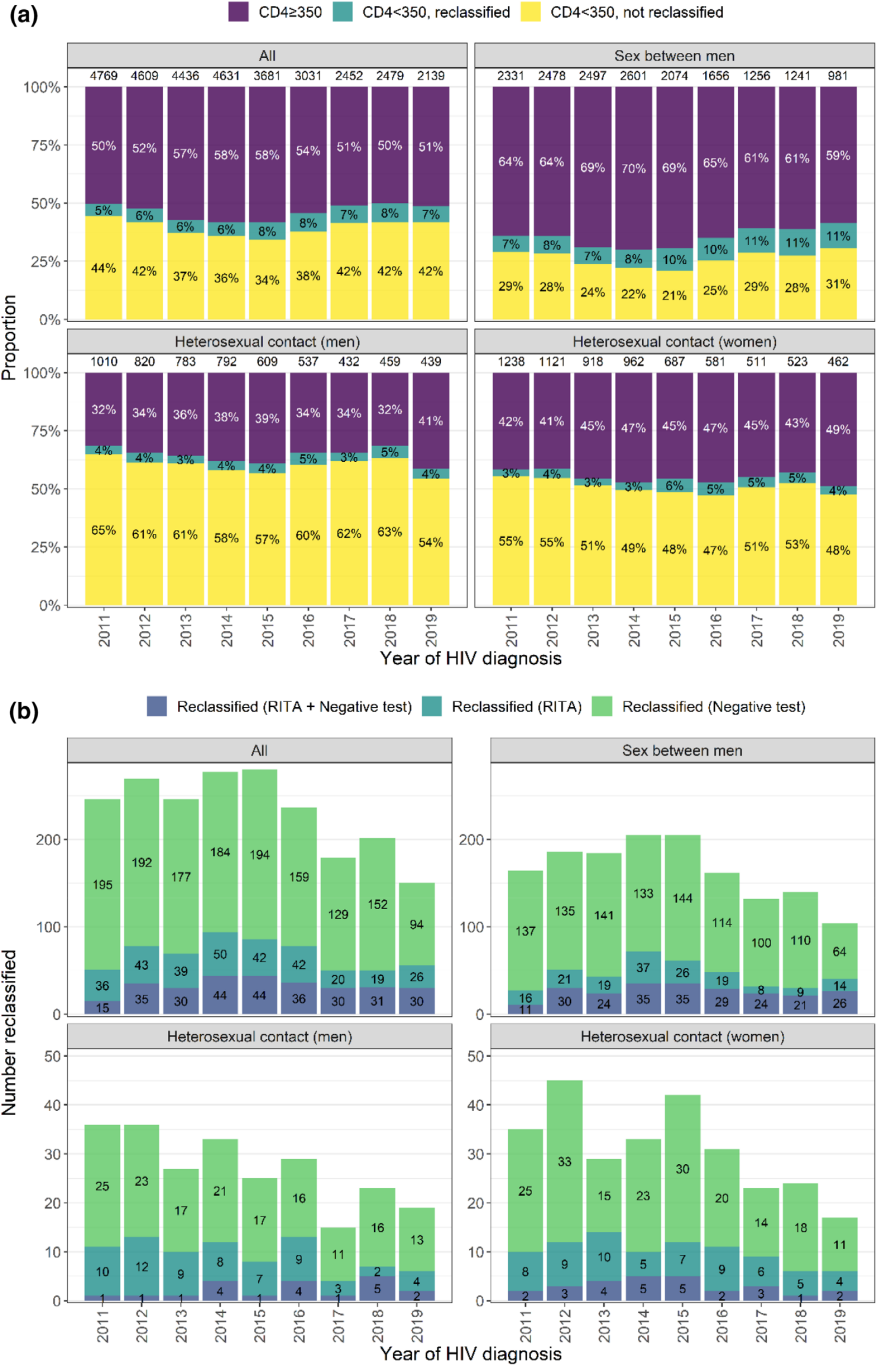
HIV diagnoses by year of HIV diagnosis, route of HIV exposure, CD4 count, re‐classification (a) and reason for re‐classification (b). Total number of diagnoses by each year and exposure group shown at top of bar in (a). Note differing axes in (b). RITA, recent infection testing algorithm

Figure 2a shows the proportion of individuals diagnosed with CD4 ≥350 cells/mm^3^ and CD4 <350 cells/mm^3^, by reclassification and probable route of HIV exposure. The evidence used to reclassify is shown in Figure 2b. Among GBM, the proportion with a CD4 <350 cells/mm^3^ fell from 36% in 2011 to 30% in 2014, rising to 41% in 2019, compared with a relatively steady reduction among heterosexual men (from 68% in 2011 to 59% in 2019) and women (from 58% in 2011 to 51% in 2019). The number and proportion reclassified was greater among GBM than heterosexual men and women, reflected in an overall CF of 25% among GBM compared with 6% and 7%, respectively (Tables 3, 4, 5).

**TABLE 3:** Late diagnosis (CD4 <350 cells/mm^3^) rates before and after correction, and correction factors, for gay and bisexual men with baseline CD4 count, by year of diagnosis, age group and region of diagnosis

	Total	CD4 <350	CD4 <350 and recent RITA	CD4 <350 and negative test within 24 months	Total reclassified^a^	Late diagnosis rate before correction^b^ (%)	Late diagnosis rate after correction^c^ (%)	Correction factor^d^ (%)
All years	17 115	5884	404	1313	1482	34	26	25
Year of diagnosis								
2011	2331	841	27	148	164	36	29	20
2012	2478	888	51	165	186	36	28	21
2013	2497	775	43	165	184	31	24	24
2014	2601	782	72	168	205	30	22	26
2015	2074	637	61	179	205	31	21	32
2016	1656	581	48	143	162	35	25	28
2017	1256	492	32	124	132	39	29	27
2018	1241	482	30	131	140	39	28	29
2019	981	406	40	90	104	41	31	26
Age group, years								
15–24	2578	649	86	262	298	25	14	46
25–34	6587	1965	175	543	614	30	21	31
35–49	5912	2196	104	392	433	37	30	20
50–64	1801	944	35	106	124	52	46	13
≥65	237	130	4	10	13	55	49	10
Region of diagnosis								
London	8432	2398	211	640	726	28	20	30
Midlands and East of England	2432	982	53	172	189	40	33	19
North of England	3112	1238	87	268	309	40	30	25
South of England	2402	947	44	194	213	39	31	22
Wales/Northern Ireland	737	319	9	39	45	43	37	14

Abbreviation: RITA, recent infection testing algorithm.

^a^ Total reclassified = CD4 <350 cells/mm^3^ and recent RITA + CD4 <350 cells/mm^3^ and negative test within 24 months.

^b^ Late diagnosed before correction = CD4 <350 cells/mm^3^/total.

^c^ Late diagnosed after correction = (CD4 <350 cells/mm^3^ − total reclassified)/total.

^d^ Correction factor = total reclassified/CD4 <350 cells/mm^3^.

**TABLE 4:** Late diagnosis rates before and after correction, and correction factors, for heterosexual men with baseline CD4 count, by year of diagnosis, age group and region of diagnosis

	Total	CD4 <350	CD4 <350 and recent RITA	CD4 <350 and negative test within 24 months	Total reclassified^a^	Late diagnosis rate before correction^b^ (%)	Late diagnosis rate after correction^c^ (%)	Correction factor^d^ (%)
All years	5881	3805	84	179	243	65	61	6
Year of diagnosis								
2011	1010	691	11	26	36	68	65	5
2012	820	539	13	24	36	66	61	7
2013	783	504	10	18	27	64	61	5
2014	792	492	12	25	33	62	58	7
2015	609	371	8	18	25	61	57	7
2016	537	353	13	20	29	66	60	8
2017	432	283	4	12	15	66	62	5
2018	459	314	7	21	23	68	63	7
2019	439	258	6	15	19	59	54	7
Age group								
15–24	275	119	7	17	22	43	35	18
25–34	1174	635	25	54	73	54	48	11
35–49	2744	1859	26	69	90	68	64	5
50–64	1401	977	21	30	46	70	66	5
65+	287	215	5	9	12	75	71	6
Region of diagnosis								
London	2090	1318	35	82	106	63	58	8
Midlands and East of England	1473	1005	14	45	56	68	64	6
North of England	1150	741	19	27	43	64	61	6
South of England	910	577	11	23	31	63	60	5
Wales/Northern Ireland	258	164	5	2	7	64	61	4

Abbreviation: RITA, recent infection testing algorithm.

^a^ Total reclassified = CD4 <350 cells/mm^3^ and recent RITA + CD4 <350 cells/mm^3^ and negative test within 24 months.

^b^ Late diagnosed before correction = CD4 <350 cells/mm^3^/total.

^c^ Late diagnosed after correction = (CD4 <350 cells/mm^3^ − total reclassified)/total.

^d^ Correction factor = total reclassified/CD4 <350 cells/mm^3^.

**TABLE 5:** Late diagnosis rates before and after correction, and correction factors, for heterosexual women with baseline CD4 count, by year of diagnosis, age group and region of diagnosis

	Total	CD4 <350	CD4 <350 and recent RITA	CD4 <350 and negative test within 24 months	Total reclassified^a^	Late diagnosis rate before correction^b^ (%)	Late diagnosis rate after correction^c^ (%)	Correction factor^d^ (%)
All years	7003	3887	90	216	279	56	52	7
Year of diagnosis								
2011	1238	722	10	27	35	58	55	5
2012	1121	657	12	36	45	59	55	7
2013	918	501	14	19	29	55	51	6
2014	962	508	10	28	33	53	49	6
2015	687	375	12	35	42	55	48	11
2016	581	306	11	22	31	53	47	10
2017	511	282	9	17	23	55	51	8
2018	523	299	6	19	24	57	53	8
2019	462	237	6	13	17	51	48	7
Age group								
15–24	638	232	11	46	50	36	29	22
25–34	2046	1031	28	84	103	50	45	10
35–49	2983	1786	33	66	91	60	57	5
50–64	1184	736	16	19	32	62	59	4
65+	152	102	2	1	3	67	65	3
Region of diagnosis								
London	2713	1509	39	101	127	56	51	8
Midlands and East of England	1787	986	18	52	64	55	52	6
North of England	1224	670	22	31	48	55	51	7
South of England	1037	593	11	29	37	57	54	6
Wales/Northern Ireland	242	129	0	3	3	53	52	2

Abbreviation: RITA, recent infection testing algorithm.

^a^ Total reclassified = CD4 <350 cells/mm^3^ and recent RITA + CD4 <350 cells/mm^3^ and negative test within 24 months.

^b^ Late diagnosed before correction = CD4 <350 cells/mm^3^/total.

^c^ Late diagnosed after correction = (CD4 <350 cells/mm^3^ − total reclassified)/Total.

^d^ Correction factor = total reclassified / CD4 <350 cells/mm^3^.

Among GBM, the CF rose from 20% in 2011 to 32% in 2015, stabilizing at around 27% in 2016–2019. The CF was highest among younger GBM (46% for those aged 15–24 years vs. 10% for ≥65 years) and elevated for GBM diagnosed in London (30%) compared with other parts of EW&NI (range 14%–25%) (Table 3).

For heterosexual men, the CF grew from 5% in 2011 to 8% in 2016, remaining around 7% in subsequent years. For heterosexual women, these figures were 5% in 2011, 11% in 2015, and around 8% in subsequent years (Tables 4 and 5). The CF was higher among younger heterosexual men and women (18% and 22%, respectively, for those aged 15–24 years, compared with 6% and 3% for ages ≥65 years) and marginally higher in London (8%) than in the rest of EW&NI (2–7%).

**FIGURE 3:**
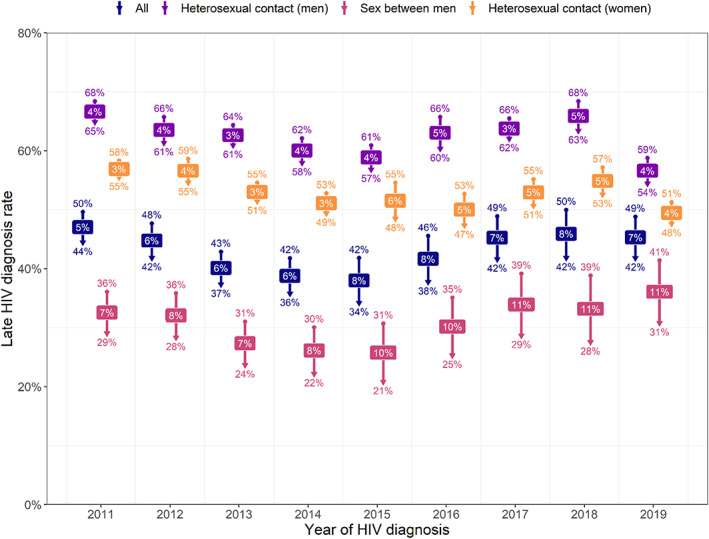
Absolute change in late diagnosis rate following reclassification, by route of exposure and year of HIV diagnosis, 2011–2019. Upper value is late diagnosis rate before reclassification, lower value is late diagnosis rate after reclassification, labels are absolute change in late diagnosis rate

Figure 3 depicts the absolute change in late diagnosis rate following reclassification. This increased over time among GBM (from 7% in 2011 to 11% in 2019), remaining relatively stable for heterosexual men and women (between 3% and 6%).

### Sensitivity analyses

By reducing the threshold for a last negative test to 12 months, 553 fewer diagnoses were reclassified, resulting in a CF of 10% and late diagnosis rate of 41% (vs. CF of 14% and 39% late diagnosed using the 24‐month threshold). The shorter threshold resulted in a CF of 19% (vs. 25%) among GBM and 5% and 5% among heterosexual men and women, respectively (vs. 6% and 7%) (Table S2).

Using solely a last negative test within the past 24 months to reclassify diagnoses resulted in a CF of 12% overall and late diagnosis rate of 40%. The CF was 22% for GBM, 5% for heterosexual men, and 6% for heterosexual women. Conversely, using solely RITA information resulted in a CF of 4% overall, 7% for GBM, and 2% for both heterosexual men and women (Table S2).

A total of 17 822 people had both a baseline CD4 count and a RITA result reported. In this group, 46% (8198) had a CD4 <350 cells/mm^3^, of whom 7% (612/8189) had a RITA result indicating recent acquisition; 13% (1092/8189) had a negative test within 24 months; and 17% (1409/8189) had at least one of these. After reclassification, the late diagnosis rate was 38% overall, with a CF of 17%. The CF was 29% for GBM, 8% for heterosexual men, and 9% for heterosexual women (Table S2).

### Risk factors for reclassification

**FIGURE 4:**
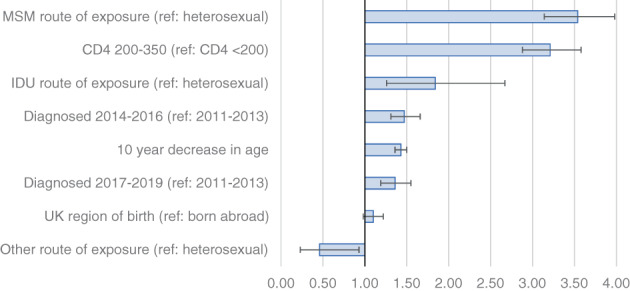
Adjusted odds ratios and 95% confidence intervals for reclassification of late diagnosis among people diagnosed with a CD4 count <350 cells/mm^3^

In multivariable analysis adjusted for other factors, the odds of being reclassified for GBM were three times those of heterosexual men and women (adjusted odds ratio [aOR] 3.54; 95% confidence interval [CI] 3.14–3.98; *p* < 0.001) and the odds for those with a baseline CD4 between 200 and 350 cells/mm^3^ were three times those with a CD4 <200 cells/mm^3^ (aOR 3.21; 95% CI 2.88–3.58; *p* < 0.001). A younger age at diagnosis (aOR 1.43; 95% CI 1.36–1.50; *p* < 0.001 for each 10‐year decrease in age) and more recent diagnosis (2014–16 and 2017–19 vs. 2011–13) were also significant factors for reclassification (Table 6/Figure 4). There was an approximately linear relationship between increasing CD4 count and the proportion of diagnoses reclassified (Figure S2).

**TABLE 6:** Logistic regression showing risk factors for reclassification among people diagnosed with a CD4 count <350 cells/mm^3^, *p*‐values shown for adjusted odds ratios

Factor	OR	95% CI	aOR	95% CI	*p*‐value
10‐year decrease in age	1.64	1.57–1.72	1.43	1.36–1.50	<0.001
Year of diagnosis					
Diagnosed 2011–2013	1.00		1.00		
Diagnosed 2014–2016	1.47	1.32–1.63	1.47	1.31–1.66	<0.001
Diagnosed 2017–2019	1.35	1.20–1.52	1.36	1.19–1.55	<0.001
CD4 count					
<200	1.00		1.00		
200–350	4.21	3.81–4.67	3.21	2.88–3.58	<0.001
Region of birth					
UK born	1.00		1.00		
Born abroad	1.52	1.38–1.67	1.10	0.98–1.22	0.1
Probable route of HIV exposure					
Heterosexual sex	1.00		1.00		
Sex between men	4.62	4.16–5.14	3.54	3.14–3.98	<0.001
Injecting drug use	1.82	1.27–2.60	1.84	1.26–2.67	0.001

Abbreviations: aOR, adjusted OR, CI, confidence interval; OR, odds ratio; RITA, recent infection testing algorithm.

### One‐year mortality

Considering individuals with a baseline CD4 count and at least 1 year of follow‐up, 158 per 10 000 population (447/30 088) diagnosed between 2011–2018 died within a year of diagnosis, of whom 89% (*n* = 396) were diagnosed with a CD4 <350 cells/mm^3^. The 1‐year mortality rate prior to correction was 288 per 10 000 for people with a CD4 <350 cells/mm^3^ compared with 31.2 per 10 000 with a CD4 ≥ 350 cells/mm^3^. Following reclassification, these rates were 329 and 31.8 per 10 000 (36.1 per 10 000 among those reclassified) (Table 7). All seven reclassified individuals who died had non‐HIV‐related causes of death. Mortality rates did not change substantially over the study period.

**TABLE 7:** One‐year mortality among people diagnosed between 2011 and 2018,^a^ by late diagnosis status before and after reclassification

	n	No. of deaths within 1 year of diagnosis	One‐year mortality rate per 10 000
Before reclassification			
Diagnosed late	13 759	396	288
Diagnosed promptly	16 329	51	31.2
After reclassification			
Diagnosed late	11 821	389	329
Diagnosed promptly	18 267	58	31.8
Among those reclassified	1938	7	36.1

Abbreviation: RITA, recent infection testing algorithm.

a Analysis considered 1‐year mortality among people diagnosed 2011–2018 with a baseline CD4 count and at least 1 year of follow‐up, i.e. deaths to the end of 2019. Deaths occurring in 2020 were not considered because of the impact of the COVID‐19 pandemic.

## DISCUSSION

The success of combination prevention initiatives, including testing campaigns [23], immediate ART initiation [36], and use of PrEP by those most at risk [37], is evident in estimated declines in incident cases and estimated reductions in undiagnosed HIV prevalence in England since 2013 [38, 39]. The proportion of people diagnosed late each year has been an important surveillance output used to direct and target these HIV‐prevention campaigns, and late diagnosis is a key indicator of the English Public Health Outcome Framework, presented by key exposure group and geography on the fingertips mapping tool [40].

In this study, we reassessed the surveillance definition of late diagnosis in the context of people testing more frequently for HIV, leading to higher likelihood of being diagnosed during seroconversion. Our analyses indicate that an increasing proportion of people diagnosed with HIV have evidence of a recent negative HIV test. We show that, when testing history is combined with knowledge of recent infection using RITA, the current public health definition of late diagnosis (CD4 <350 cells/mm^3^) increasingly overestimates rates of late presentation. Reclassification for seroconversion resulted in a downwards adjustment of the late diagnosis rate by 14% in 2019. This was highest among GBM (26% in 2019), particularly younger GBM (CF of 46% for those aged 15–24 years) and those living in London (CF of 30%), which may reflect the concentration of HIV‐prevention campaigns and high testing volumes at SHS (and therefore higher likelihood of being diagnosed during seroconversion) in this age group and region than in other areas of the country [41, 42, 43]. Meanwhile, groups for whom reclassification was less common (e.g. older people and those who likely acquired HIV through heterosexual sex) are known to have lower testing rates [21].

For GBM, the CF increased between 2011 and 2015 (from 20% to 32%), declining to around 27% in more recent years despite high testing rates being maintained. The recent decline may reflect the reduction in HIV incidence among GBM estimated in recent years [38]. The knock‐on effect is an upward trend in late diagnosis rates (from 21% in 2015 to 31% in 2019, after accounting for seroconversion). We surmise that fewer HIV infections acquired (e.g. due to PrEP) led to a smaller population able to be diagnosed at an early stage, thereby shrinking the denominator for the late diagnosis indicator more rapidly than the numerator. Meanwhile, modest declines in late diagnosis rates were observed for heterosexuals. Unlike in GBM, frequent testing and uptake of PrEP among higher‐risk heterosexuals has not been observed [21, 44], hence fewer heterosexuals were diagnosed during seroconversion, resulting in a comparatively low CF. Trends in late diagnosis rates must be interpreted alongside the absolute number of diagnoses, as well as estimated incidence, testing, and PrEP use.

In an era of effective treatments, late presentation of HIV is the strongest predictor of premature death [14, 15]. In our study, people diagnosed with a CD4 <350 cells/mm^3^ had a nine‐fold increased risk of death within a year of diagnosis compared with those with a CD4 ≥ 350 cells/mm^3^, which increased to 10‐fold after reclassification.

Sasse et al. used evidence of recent acquisition within 6 months, clinical presentation with acute infection, and/or recent risk behaviour with a HIV‐positive partner to reclassify late diagnoses [28]. While our definition was less stringent, we consider it more appropriate for EW&NI as it includes less frequent testers and those without testing history. For people with both a recent RITA and testing history, the median time from previous negative test to diagnosis was 6 months, with 86% of previous negative tests within 24 months. Use of testing history alone could be a pragmatic approach to correct for seroconversion in countries lacking detailed clinical or serological information. Where collected, information from diagnostic tests could also provide a useful proxy for recency, as recent studies have shown correlation between a ‘recent’ limiting antigen result and a signal‐to‐cut‐off ratio <250 in the widely‐used Abbott Architect Ag/Ab assay [45, 46].

The strengths of this study are the very high coverage of HIV diagnoses, high completeness of key variables, and long period over which we could link data. A limitation was differential geographical coverage of RITA results, which may have biased reclassification towards certain regions. Additionally, HIV testing history outside SHS was poorly captured; whereas GBM are more likely to attend SHS, heterosexual men and women may access testing in a wider variety of settings [21].

In this study, we focused on those with a first diagnosis in EW&NI as we could not capture ART initiation or engagement with care prior to arrival. In 2019, 72% of those previously diagnosed abroad were virally suppressed, with only 22% having a CD4 <350 cells/mm^3^ at first presentation in EW&NI (compared with 49% for the study population). Although this group was not included in our analysis, the health needs of migrants remain a public health priority, especially given the potential for disengagement in care following relocation.

## CONCLUSIONS

As highlighted in the recent English HIV Action Plan [11], late diagnosis remains a priority area in HIV case‐reporting surveillance. It provides an indicator of a ‘system failure’ to promptly diagnose, provide benefit through ART, and reduce onward transmission and has the advantage of being relatively easy to measure. Tackling barriers that may lead to late presentation, such as testing access, lack of risk awareness, and HIV‐related stigma, is vital to reduce the number of late diagnoses [47, 48].

Our refined definition of late HIV diagnosis focuses this measure on those at greatest risk of death. This is ever‐more important as the overall number of diagnoses reduces, since unadjusted rates may present a misleading picture of where late diagnoses are occurring, undermining targeted interventions tailored towards late diagnosis. This refinement is also key for detailed incident follow‐up of individual cases and may contribute to the development of statistical models that use CD4 count information to estimate time since infection [7, 38, 49]. For example, inclusion of RITA and/or last negative test information as biomarkers in these models may aid model performance and help to address misclassification of long‐standing infection.

## AUTHOR CONTRIBUTIONS

Valerie C Delpech, Alison E Brown, and Peter D Kirwan conceived the research study. Jennifer Tosswill and Gary Murphy performed the RITA testing. Adamma Aghaizu implemented the RITA algorithm. Peter D Kirwan ran the RITA algorithm and data linkage and performed the analysis. Peter D Kirwan, Sara Croxford, and Valerie C Delpech drafted and finalized the manuscript. All authors critically appraised the manuscript and approved its submission.

## CONFLICT OF INTEREST

All authors have completed the unified competing interest form (available on request from the corresponding author) and declare no support for the submitted work from anyone other than their employer, no financial relationships with any organizations that might have an interest in the submitted work in the previous 3 years, and no other relationships or activities that could appear to have influenced the submitted work.

## ETHICS STATEMENT

HIV surveillance data were collected by the UKHSA with permissions granted under Regulation 3 of The Health Service (Control of Patient Information) Regulations 2002, and without explicit permission under Section 251 of the NHS Act 2006. All datasets are held securely at UKHSA, where strict confidentiality is maintained. Names and NHS numbers are not collected. The research study was classified as surveillance undertaken as part of UKHSA's legal responsibility to monitor HIV and exempted from full ethical review by the UKHSA Research Support and Governance Office.

## Supporting information


**Appendix S1** Supporting Information.

## Data Availability

The data used in this study are protected data. These data are not publicly available because the information is personal or special category personal data, and there is risk of ‘re‐identification’ of data that have been anonymised by data matching, inference, or deductive disclosure. Access to protected data is subject to robust governance protocols, where it is lawful, ethical, and safe to do so. Individuals and organizations wishing to request access to data used in this study, can make a request directly to UKHSA (https://www.gov.uk/government/publications/accessing-ukhsa-protected-data). Access to protected data is always strictly controlled using legally binding data‐sharing contracts.
