# Supplementary material for: Re‐assessing the late HIV diagnosis surveillance definition in the era of increased and frequent testing
Source: HIV Med. 2022 Sep 7;23(11):1127–42. doi: 10.1111/hiv.13394 (PMC7613879; doi:10.1111/hiv.13394)
Supplement: Supplementary file 1 — Appendix S1 Supporting Information. [file HIV-23-1127-s001.docx]

**Appendix**

*Biomarker assays and RITA definition*

The biomarker assays used were the HIV 1/2gO AxSYM assay (Abbott Laboratories, USA) [1] for samples prior to 2014 and Sedia LAg assay (Sedia Biosciences Corporation, USA) [2] for samples tested from 2014 onwards.

The Recent Infection Testing Algorithm (RITA) defines a ‘recent’ diagnosis as an AxSYM Avidity index score less than 80.0% or a LAg normalised optical density score less than 1.5 within 120 days of HIV diagnosis and neither of: a CD4 count <50 cells/mm^3^, an AIDS diagnosis, nor an HIV viral load<400 copies/mL within 90 days of HIV diagnosis. Otherwise the diagnosis is classified as not recent.

A ‘recent’ RITA result indicated probable HIV acquisition within a mean of either 6 (AxSYM) or 4 (LAg) months of HIV diagnosis, with benchmarking of the two assays previously undertaken to ensure consistency over time [3,4]. Tests were excluded from RITA calculations if the date of the sample was more than 120 days since diagnosis or if the test was after ART initiation (n=924) (Supplementary Figure 1).

References:

1. Suligoi B, Galli C, Massi M, Di Sora F, Sciandra M, Pezzotti P, et al. Precision and accuracy of a procedure for detecting recent human immunodeficiency virus infections by calculating the antibody avidity index by an automated immunoassay-based method. Journal of clinical microbiology. 2002;40(11):4015-20.
2. Sedia Biosciences Corporation. Sedia HIV-1 LAg-Avidity EIA: single well avidity enzyme immunoassay for detection of recent HIV-1 infection using liquid serum or plasma, Cat. No. 1002 2013. [cited 7 Jan 2022] Available: www.sediabio.com/LiteratureRetrieve.aspx?ID=139682
3. Kassanjee R, Pilcher CD, Keating SM, Facente SN, McKinney E, Price MA, et al. Independent assessment of candidate HIV incidence assays on specimens in the CEPHIA repository. AIDS. 2014;28(16):2439-49.
4. Murphy G, Parry JV. Assays for the detection of recent infections with human immunodeficiency virus type 1. Euro Surveillance: Bulletin Europeen sur les Maladies Transmissibles = European Communicable Disease Bulletin. 2008;13(36).

**Supplementary Figure 1: Recent Infection Testing Algorithm (RITA) flowchart**

New HIV diagnosis

No serological test or >120 days

Serological test within 120 days

Excluded

Avidity^1^≥80 or LAg^2^≥1.5

Avidity^1^<80 or LAg^2^<1.5

Not recent

Treatment before sample

CD4<50 or AIDS within 3 months or VL<400

Excluded

Recent

Not recent

Footnotes:

1. Avidity = AxSYM avidity assay.

2. LAg = Sedia LAg assay.

**Supplementary Figure 2: CD4 count distribution for reclassified and non-reclassified diagnoses with CD4<350, by probable route of exposure and CD4 count**


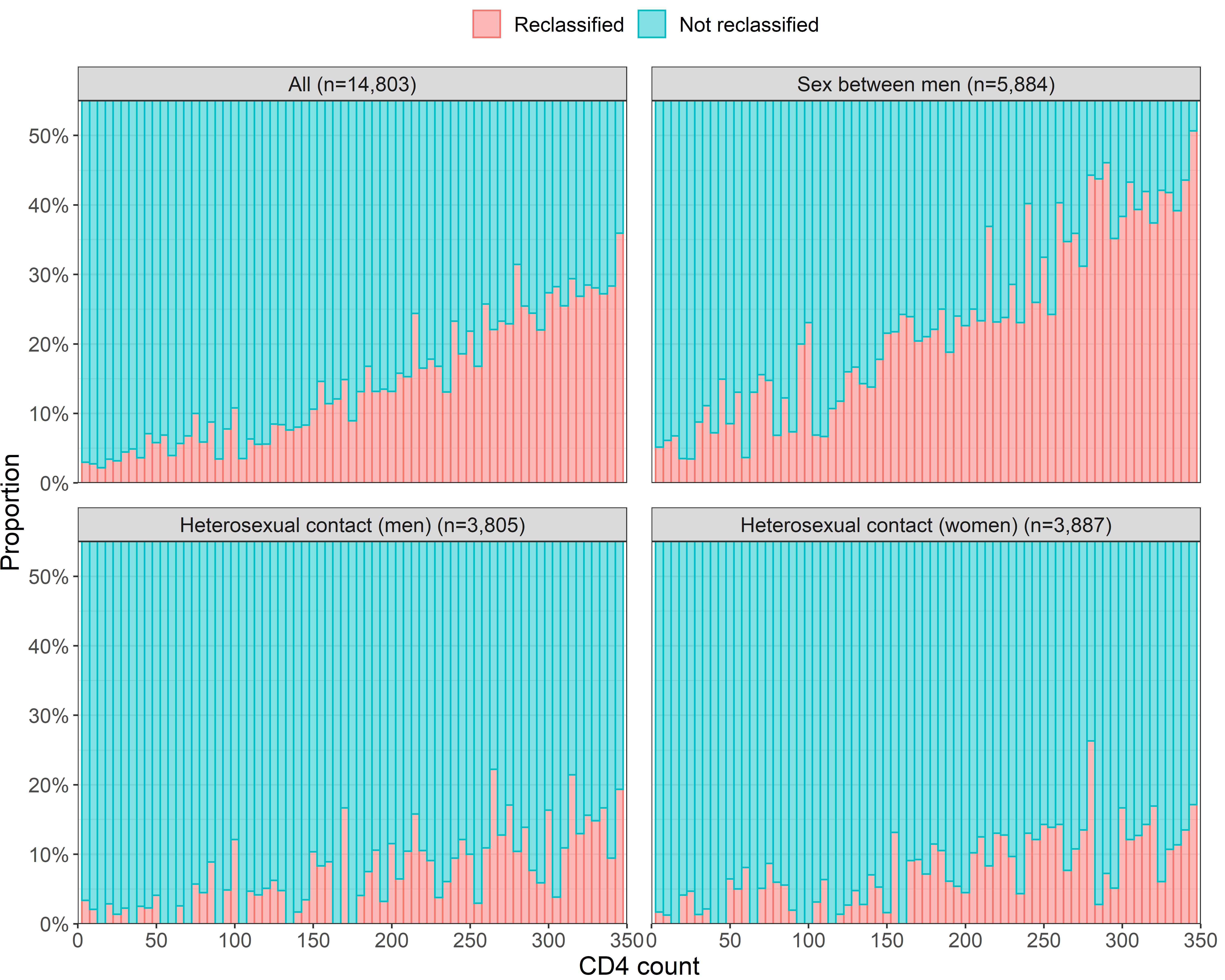


**Supplementary Figure 3: HIV diagnoses among those with RITA result recorded (i.e. sensitivity analysis 4), by year of HIV diagnosis, route of HIV exposure, CD4 count, re-classification (panel A) and reason for re-classification (panel B)**


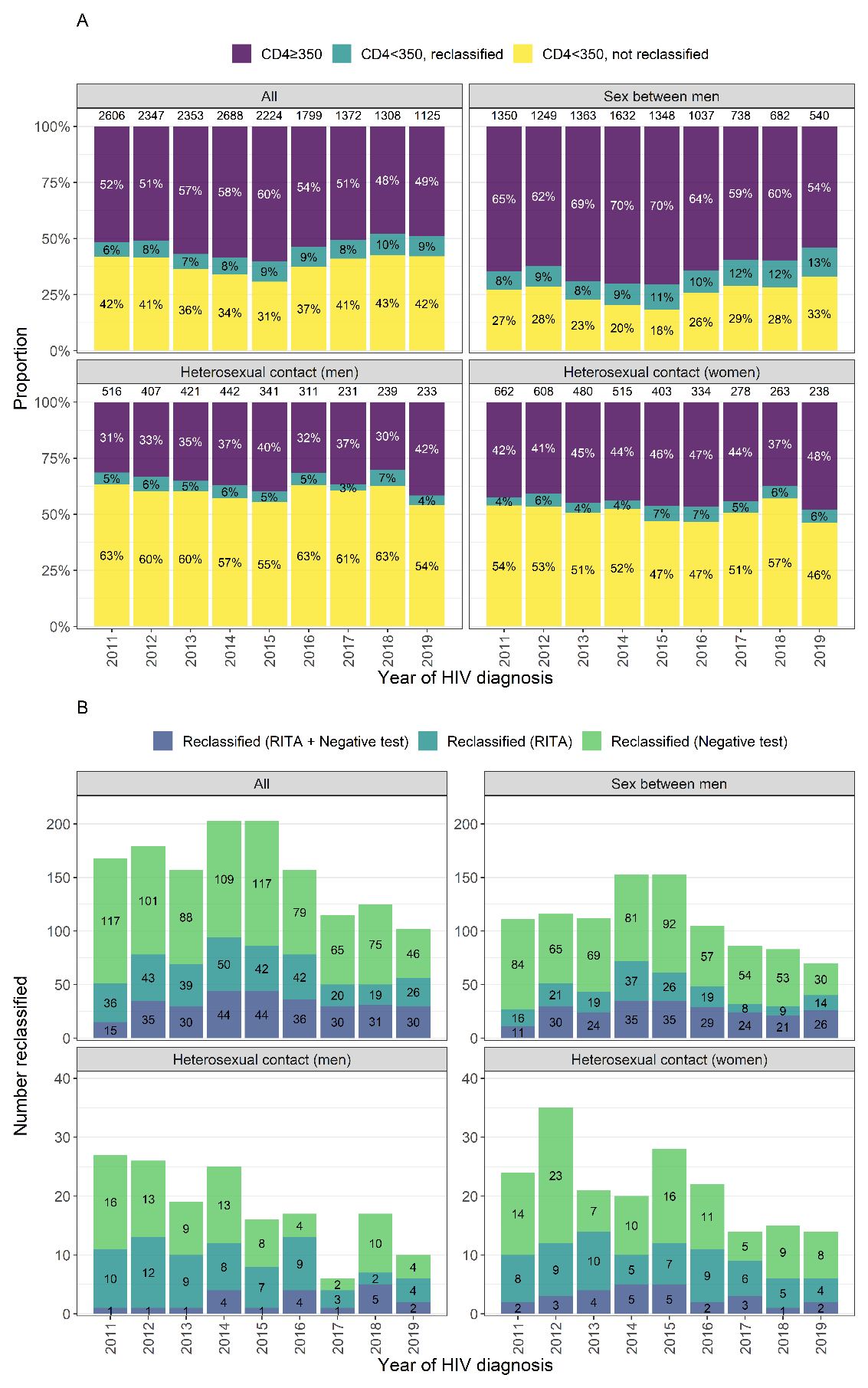


Footnotes:

1. Total number of diagnoses by each year and exposure group shown at top of bar in panel A.

2. Note differing axes in panel B.

**Supplementary table 1: Total number of individuals diagnosed, number and proportion included in study population, number and proportion with a RITA result or negative HIV test available, and number and proportion tested for recent infection (prior to application of RITA algorithm)**

|  | Number diagnosed | Study population^1^ | | RITA result or negative HIV test available | | Tested for recent infection | |
| --- | --- | --- | --- | --- | --- | --- | --- |
|  | n | n | % | n | %^2^ | n | %^2^ |
| All years | 39,909 | 32,227 | 81% | 22,993 | 71% | 18,746 | 58% |
| **Year of HIV diagnosis** |  |  |  |  |  |  |  |
| 2011 | 5,408 | 4,769 | 88% | 3,220 | 68% | 2,764 | 58% |
| 2012 | 5,441 | 4,609 | 85% | 3,033 | 66% | 2,485 | 54% |
| 2013 | 5,251 | 4,436 | 84% | 3,101 | 70% | 2,492 | 56% |
| 2014 | 5,497 | 4,631 | 84% | 3,385 | 73% | 2,809 | 61% |
| 2015 | 4,771 | 3,681 | 77% | 2,828 | 77% | 2,330 | 63% |
| 2016 | 3,903 | 3,031 | 78% | 2,316 | 76% | 1,901 | 63% |
| 2017 | 3,407 | 2,452 | 72% | 1,805 | 74% | 1,442 | 59% |
| 2018 | 3,288 | 2,479 | 75% | 1,768 | 71% | 1,372 | 55% |
| 2019 | 2,943 | 2,139 | 73% | 1,537 | 72% | 1,151 | 54% |
| **Gender** |  |  |  |  |  |  |  |
| Male | 29,750 | 24,546 | 83% | 18,040 | 73% | 14,521 | 59% |
| Female | 10,154 | 7,676 | 76% | 4,950 | 64% | 4,224 | 55% |
| **Probable route of HIV exposure** |  |  |  |  |  |  |  |
| Sex between men | 19,464 | 17,115 | 88% | 13,544 | 79% | 10,567 | 62% |
| Heterosexual men | 6,911 | 5,881 | 85% | 3,706 | 63% | 3,258 | 55% |
| Heterosexual women | 8,263 | 7,003 | 85% | 4,614 | 66% | 3,920 | 56% |
| Injecting drug use | 745 | 554 | 74% | 342 | 62% | 283 | 51% |
| Other | 435 | 323 | 74% | 145 | 45% | 135 | 42% |
| Undetermined | 4,091 | 1,351 | 33% | 642 | 48% | 583 | 43% |
| **Country of birth** |  |  |  |  |  |  |  |
| UK born | 17,609 | 15,056 | 86% | 11,600 | 77% | 9,061 | 60% |
| Rest of Europe | 5,264 | 4,539 | 86% | 3,236 | 71% | 2,720 | 60% |
| Africa | 8,530 | 7,140 | 84% | 4,565 | 64% | 3,978 | 56% |
| Rest of World | 4,874 | 4,196 | 86% | 2,977 | 71% | 2,459 | 59% |
| Unknown | 3,632 | 1,296 | 36% | 615 | 47% | 528 | 41% |
| **Age at HIV diagnosis** |  |  |  |  |  |  |  |
| 15-24 | 4,722 | 3,816 | 81% | 2,901 | 76% | 2,380 | 62% |
| 25-34 | 12,679 | 10,396 | 82% | 7,825 | 75% | 6,269 | 60% |
| 35-49 | 15,353 | 12,474 | 81% | 8,700 | 70% | 7,069 | 57% |
| 50-64 | 5,982 | 4,773 | 80% | 3,125 | 65% | 2,648 | 55% |
| 65+ | 1,173 | 768 | 65% | 442 | 58% | 380 | 49% |
| **Region of diagnosis** |  |  |  |  |  |  |  |
| London | 17,232 | 14,215 | 82% | 10,493 | 74% | 8,962 | 63% |
| Midlands and East of England | 7,792 | 6,200 | 80% | 4,025 | 65% | 3,134 | 51% |
| North of England | 7,294 | 5,826 | 80% | 4,459 | 77% | 3,653 | 63% |
| South of England | 5,666 | 4,622 | 82% | 3,289 | 71% | 2,499 | 54% |
| Northern Ireland | 684 | 580 | 85% | 459 | 79% | 464 | 80% |
| Wales | 1,241 | 784 | 63% | 268 | 34% | 34 | 4% |

Footnotes:

^1^ Study population are individuals with a baseline CD4 count available.

^2^ Percentages are proportions of study population with RITA/Last negative result or tested for recent infection.

**Supplementary table 2: Results of sensitivity analyses 1) reducing last negative test threshold to 12 months, 2) using only last negative test data, 3) using only RITA result, 4) among population with baseline CD4 count and RITA result, by year of diagnosis, age group and region of diagnosis**

|  | Total | CD4<350 | CD4<350 and recent RITA | CD4<350 and negative test within 12 months | CD4<350 and negative test within 12-24 months | **Total reclassified^1^** | Late diagnosis rate before correction^2^ | Late diagnosis rate after correction^3^ | Correction factor^4^ |
| --- | --- | --- | --- | --- | --- | --- | --- | --- | --- |
| **Sensitivity analysis 1**  **Population:** CD4<350 **Reclassification:** recent RITA or negative test within 12 months | | | | | | | | | |
| All | 32,227 | 14,803 | 612 | 1,161 |  | 1,535 | 46% | 41% | 10% |
| Gay and bisexual men | 17,115 | 5,884 | 404 | 892 |  | 1,097 | 34% | 28% | 19% |
| Heterosexual men | 5,881 | 3,805 | 84 | 101 |  | 173 | 65% | 62% | 5% |
| Heterosexual women | 7,003 | 3,887 | 90 | 127 |  | 198 | 56% | 53% | 5% |
| **Sensitivity analysis 2**  **Population:** CD4<350 **Reclassification:** negative test within 24 months | | | | | | | | | |
| All | 32,227 | 14,803 |  | 1,161 | 610 | 1,771 | 46% | 40% | 12% |
| Gay and bisexual men | 17,115 | 5,884 |  | 892 | 421 | 1,313 | 34% | 27% | 22% |
| Heterosexual men | 5,881 | 3,805 |  | 101 | 78 | 179 | 65% | 62% | 5% |
| Heterosexual women | 7,003 | 3,887 |  | 127 | 89 | 216 | 56% | 52% | 6% |
| **Sensitivity analysis 3**  **Population:** CD4<350 **Reclassification:** recent RITA | | | | | | | | | |
| All | 32,227 | 14,803 | 612 |  |  | 612 | 46% | 44% | 4% |
| Gay and bisexual men | 17,115 | 5,884 | 404 |  |  | 404 | 34% | 32% | 7% |
| Heterosexual men | 5,881 | 3,805 | 84 |  |  | 84 | 65% | 63% | 2% |
| Heterosexual women | 7,003 | 3,887 | 90 |  |  | 90 | 56% | 54% | 2% |
| **Sensitivity analysis 4**  **Population:** CD4<350 and RITA result **Reclassification:** recent RITA or negative test within 24 months (as for main analyses) | | | | | | | | | |
| All | 17,822 | 8,198 | 612 | 696 | 396 | 1,409 | 46% | 38% | 17% |
| Gay and bisexual men | 9,939 | 3,453 | 404 | 549 | 271 | 989 | 35% | 25% | 29% |
| Heterosexual men | 3,141 | 2,043 | 84 | 48 | 51 | 163 | 65% | 60% | 8% |
| Heterosexual women | 3,781 | 2,133 | 90 | 73 | 57 | 193 | 56% | 51% | 9% |

Footnotes:

^1^ Total reclassified = CD4<350 and recent RITA + CD4<350 and negative test within 12 months + CD4<350 and negative test within 12-24 months (depending on sensitivity analysis)

^2^ Late diagnosed before correction = CD4<350 / Total

^3^ Late diagnosed after correction = (CD4<350 - Total reclassified) / Total

^4^ Correction factor = Total reclassified / CD4<350

**Supplementary table 3: Correction factors for all individuals with baseline CD4 count and RITA result (i.e. sensitivity analysis 4), by year of diagnosis, probable route of exposure, age group and region of diagnosis**

|  | All | Gay and bisexual men | Heterosexual men | Heterosexual women |
| --- | --- | --- | --- | --- |
| All years | 17% | 29% | 8% | 9% |
| **Year of diagnosis** |  |  |  |  |
| 2011 | 13% | 23% | 8% | 6% |
| 2012 | 16% | 25% | 10% | 10% |
| 2013 | 15% | 26% | 7% | 8% |
| 2014 | 18% | 31% | 9% | 7% |
| 2015 | 23% | 38% | 8% | 13% |
| 2016 | 19% | 28% | 8% | 12% |
| 2017 | 17% | 29% | 4% | 9% |
| 2018 | 18% | 30% | 10% | 9% |
| 2019 | 18% | 28% | 7% | 11% |
| **Age group** |  |  |  |  |
| 15-24 | 38% | 47% | 22% | 25% |
| 25-34 | 25% | 36% | 14% | 12% |
| 35-49 | 13% | 22% | 6% | 7% |
| 50-64 | 9% | 15% | 6% | 6% |
| 65+ | 8% | 13% | 8% | 6% |
| **Region of diagnosis** |  |  |  |  |
| London | 21% | 35% | 9% | 10% |
| Midlands and East of England | 12% | 21% | 7% | 9% |
| North of England | 15% | 24% | 7% | 9% |
| South of England | 17% | 26% | 9% | 8% |
| Wales/Northern Ireland | 12% | 19% | 8% | 3% |
